# Analysis of Thyroid Response Element Activity during Retinal Development

**DOI:** 10.1371/journal.pone.0013739

**Published:** 2010-10-29

**Authors:** Nathan A. Billings, Mark M. Emerson, Constance L. Cepko

**Affiliations:** 1 Department of Genetics, Harvard Medical School, Boston, Massachusetts, United States of America; 2 Howard Hughes Medical Institute, Department of Genetics, Harvard Medical School, Boston, Massachusetts, United States of America; University of Colorado, Boulder, United States of America

## Abstract

Thyroid hormone (TH) signaling components are expressed during retinal development in dynamic spatial and temporal patterns. To probe the competence of retinal cells to mount a transcriptional response to TH, reporters that included thyroid response elements (TREs) were introduced into developing retinal tissue. The TREs were placed upstream of a minimal TATA-box and two reporter genes, green fluorescent protein (GFP) and human placental alkaline phosphatase (PLAP). Six of the seven tested TREs were first tested *in vitro* where they were shown to drive TH-dependent expression. However, when introduced into the developing retina, the TREs reported in different cell types in both a TH-dependent and TH-independent manner, as well as revealed specific spatial patterns in their expression. The role of the known thyroid receptors (TR), TRα and TRβ, was probed using shRNAs, which were co-electroporated into the retina with the TREs. Some TREs were positively activated by TR+TH in the developing outer nuclear layer (ONL), where photoreceptors reside, as well as in the outer neuroblastic layer (ONBL) where cycling progenitor cells are located. Other TREs were actively repressed by TR+TH in cells of the ONBL. These data demonstrate that non-TRs can activate some TREs in a spatially regulated manner, whereas other TREs respond only to the known TRs, which also read out activity in a spatially regulated manner. The transcriptional response to even simple TREs provides a starting point for understanding the regulation of genes by TH, and highlights the complexity of transcriptional regulation within developing tissue.

## Introduction

Appropriate levels of TH are critical for the proper development and maturation of several tissues, including the brain [1]–[8], cochlea [9]–[13], and retina [14]–[17]. TRs are nuclear hormone transcription factors that bind specific DNA sequences (TREs) and modulate gene transcription in a ligand-dependent bimodal fashion. Heterodimerization with a retinoid X receptor (RXR) enhances binding of some nuclear receptors (NRs), including the Vitamin D3 Receptors (VDRs), TRs, and Retinoic Acid Receptors (RARs), to their respective response elements [18]–[20]. Many consensus TREs consist of various permutations of a half site, AGGTCA, as the core motif sequence [21]. Most NRs of the estrogen-like superfamily bind this half site efficiently. However ligand-specificity is conferred via the spacing and orientation of each half site repeat. One well characterized T3-specific TRE is the direct repeat +4 element (DR4), consisting of the sequence AGGTCA*nnnn*AGGTCA. Adding or subtracting one base from the interval between the two direct repeats diminishes T3 ligand sensitivity and introduces sensitivity to either retinoic acid (RA) with a 5 nucleotide spacer (DR+5) or Vitamin D3 (VitD3) with a 3 nucleotide spacer (DR+3) [22].

TH target gene recognition *in vivo* is more complex than just a single TH-sensitive TRE in the promoter. It is the result of the complex interactions of multiple transcription factors binding promoter elements and enhancers, as well as multiple dynamic signaling pathways competing for heterodimer partners and co-factors [23]–[27]. For example, RARs and TRs are able to bind some of the same cofactors, providing for a layer of regulation which likely integrates the spatial and temporal levels of the respective ligands as well as their receptors [26], [27]. Competing factors, such as the COUP-TFs, display restricted spatial patterns of expression across the retina, as well as varied expression in different retinal cell types in one location [28], [29]. COUP-TFs bind a variety of direct repeats and are thought to repress TH and RA signaling through competition for occupancy of TREs and competition for RXR, providing yet another layer of regulation [23]–[25].

A very specific level of regulation of TH signaling is through a class of selenocysteine proteins named the deiodinases. Type 2 deiodinase (Dio2) is required for tissue-specific activation of the pro-hormone, thyroxine (T4), to 3,5,3'-triiodothyronine (T3), whereas type 3 deiodinase (Dio3) deiodinates both T4 and T3 to biologically inactive metabolites [30]–[32]. Although deiodinase activity provides a simple mechanism by which to control hormone exposure, both Dio2 and Dio3 have been shown to be controlled by multiple additional signaling pathways. In rats for example, Dio3 is activated in hypoxic tissue by HIF-1a [33]. In keratinocytes, Sonic hedgehog (Shh) activates Dio3 and enhances proliferation, and in chicken tibial explants, Shh inactivates Dio2 [34], [35]. In the rodent brain, tissue-specific deiodination via Dio2 is required to produce T3 for the entire tissue [36]. In avian species, the majority of TH deposited in the yolk is in the inactive form of T4, and thus tissue-specific deiodination is required for proper development [37]–[40].

TH signaling components, consisting of the thyroid hormone receptors (TRs), TRα and TRβ, as well as the deiodinases, Dio2 and Dio3, are expressed in unique spatial and temporal patterns in the developing avian retina [41]. A wave of Dio2 RNA expression was observed to follow a unique temporal and spatial pattern during early chick retinal development. This wave was followed by a wave of Dio3 RNA expression. Interestingly, Dio2 and Dio3 RNA expression was detected in different cell types. Dio2 RNA was observed in a subset of postmitotic cells in the developing outer nuclear layer (ONL), in a subset of photoreceptors, whereas Dio3 RNA was observed in a subset of mitotic progenitor cells. Dio2 and TRβ2 RNAs were seen to co-localize to a small number of cells, while a larger number of postmitotic cells were only positive for either TRβ2 *or* Dio2 RNA. In the mouse, both active TH (T3) and TRβ2 are required for proper blue and green cone opsin patterning during mammalian retinal development [14], [42]–[45]. Similarly, salmonoid fish undergo a TH-dependent UV-to-blue opsin switch mediated through TRβ during development [46]–[49]. In the Drosophila retina, the Ecdysone receptor (EcR) is analogous to vertebrate TRs and functions autonomously to mediate a blue-to-green opsin switch during metamorphosis [50].

While the molecular mechanism(s) of opsin regulation by TH in vertebrates is currently being elucidated, the role of TH signaling in the earlier stages of retinal development is far less well understood. The dynamic temporal patterns of Dio2 and Dio3 RNA might suggest a “pulse-chase” of TH signaling during chick retinal development. However, it is not clear which cell type is responding to TH. Dio2, Dio3, and TRβ are for the most part located in different cell populations. TRα is expressed in most, or perhaps all cells, and thus has the potential to signal broadly. These distinct localizations of the signaling components, as well as the dynamic nature of their expression patterns, raise the intriguing question of when and where TH signaling is actually taking place. The question of TH signaling is of interest in other developing CNS tissues as well, where Dio2 activity is present in glia, and TRs and Dio3 are both present in neurons, making the precise spatial and temporal locale of TH signaling less than clear [51]. To understand these unique patterns in the retina, we wished to investigate the competence of retinal cells to respond to TH at different times during development.

Due to the complexity of the multiple layers of regulation and dynamic signaling pathways involved, as well as the disparity in TR and deiodinase localization during chick retinal development, we chose to first ask a basic question of competence – that is, to determine which cells simply possess TRs as well as all the necessary co-factors required to respond to TH. For example, it is presently unclear if a Dio2+ cell, which expresses low levels of TRα, is competent to respond to TH, or if Dio2-mediated TH activation serves a paracrine role for the neighboring population of TRβ cells [41]. Similarly, in the rodent brain, Dio2 is produced by astrocytes whereas neurons express both TRs and Dio3, and an unresolved long-standing question in brain biology has been whether glial-derived T3 is able to reach neurons [51]–[57].

To visualize where and when retinal cells are competent to respond to T3 (although don't necessarily do so), we took advantage of previously characterized TREs, which when taken out of the natural gene context, still confer a highly specific TH response. We used the efficient method of electroporation of reporter plasmids into developing retinal tissue to probe the activity of these TREs. As well, we used co-electroporated shRNA plasmids to ask if the known TRs were responsible for any responses that were observed. We hypothesized that isolated TREs could be used to probe the competence of retinal cells to respond to T3, following addition of T3 to retinal organ cultures. Using isolated TREs rather than TH-responsive promoters substantially reduced the complexity that multiple binding sites would present. It theoretically distilled the system down to the ability of a single TR heterodimer to bind a TRE and recruit the correct co-factors (if present in the cell) in developing cells to drive TH-responsive transcription.

The TREs were placed upstream of a minimal promoter and electroporated into the developing retina. Historically, TRE activity has been evaluated by expression of sensitive enzymatic reporters, such as chloramphenicol acetyltransferase (CAT) or, more recently, luciferase. These TREs were placed upstream of promoter elements, such as the herpes simplex virus (HSV) thymidine kinase (TK) or simian virus (SV40) promoter, both of which contain various binding sequences and confer basal levels of activity in the absence of an upstream TRE [58]. However, the evaluation of TRE activity in tissue required both minimal promoter activity in the absence of a TRE, as well as a reporter signal that, unlike luciferase or CAT, could be detected such that cell type specificity could be observed.

To this end, the Stagia3 (Stop TAta eGfp Ires Ap version 3) vector consisting of a 15 bp minimal TATATAA element was used to directly drive GFP, which was followed by an IRES element and PLAP. Seven unique TREs, including the canonical DR4 element, that have been well characterized in cell culture experiments, were utilized. This approach allowed us to visualize cell-specific TR-dependent activity in the developing retina with a high degree of spatial and cellular resolution. This system not only confirmed the predicted T3-dependent activity of the developing photoreceptor layer (inferred from other species) in the chick, but illuminated the previously under-appreciated dynamic, complex, and competing nature of nuclear receptor signaling. These genetic tools, combined with knock-down of each TH component, will provide a powerful tool for unraveling the complexities of *in vivo* TH homeostasis, autonomous versus non-autonomous signaling, and early TR-dependent developmental events.

## Results

### Constructing Thyroid Response Element (TRE) reporters

**Figure 1:**
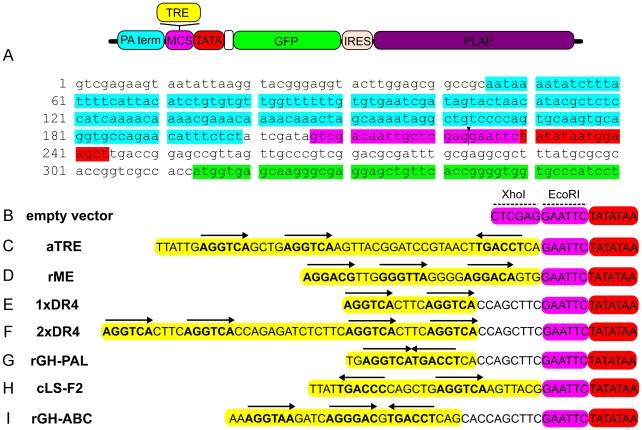
Reporter backbone and T3 response elements. A. Stagia3 backbone consisting of a minimal 15 base TATA-box driving a GFP-IRES-PLAP (human placental alkaline phosphatase) reporter cassette. A TRE (yellow) upstream of the TATA (red) should be sufficient to drive both GFP (green) and PLAP (purple). PolyA terminator sequence is denoted in aqua, the MCS is colored pink. The closed black arrow indicates the XhoI/EcoRI site of TRE insertion. B. Schematic of proximal 5′ sequence (relative to TATA) of reporter backbone. C-I. Proximal 5′ sequence (relative to TATA) of TRE reporters. Reporter constructs were constructed using TRE sequences (denoted in yellow) previously identified [22], [59]–[67]. Arrows are placed over hexamers with 4/6 bases matching the optimal AGGTCA 1/2 site consensus.

We first examined existing literature for previously described TREs that had been characterized *in vitro* for TR binding and TH activation [22], [59]–[67]. Reporters for TH were built by annealing short synthesized oligo's for each TRE, and then cloning each one into a reporter vector (Stagia3) consisting of a minimal TATA-box driving GFP-IRES-PLAP (Fig. 1A). By using small defined TREs rather than entire promoter regions from TH-responsive genes, we reasoned that it would be possible to capture only a single NR complex, and thus reduce the complexity that multiple NR and transcription factor binding sites within promoter and enhancer regions of any given gene would present. Therefore, theoretically the reporter would be active only when all the components required for activation were present, including ligand, co-receptors, and a complete co-activation complex.

### TREs upstream of minimal TATA are sufficient to drive T3-dependent reporter activity in 293T cells

#### Control vector in cell culture

**Figure 2:**
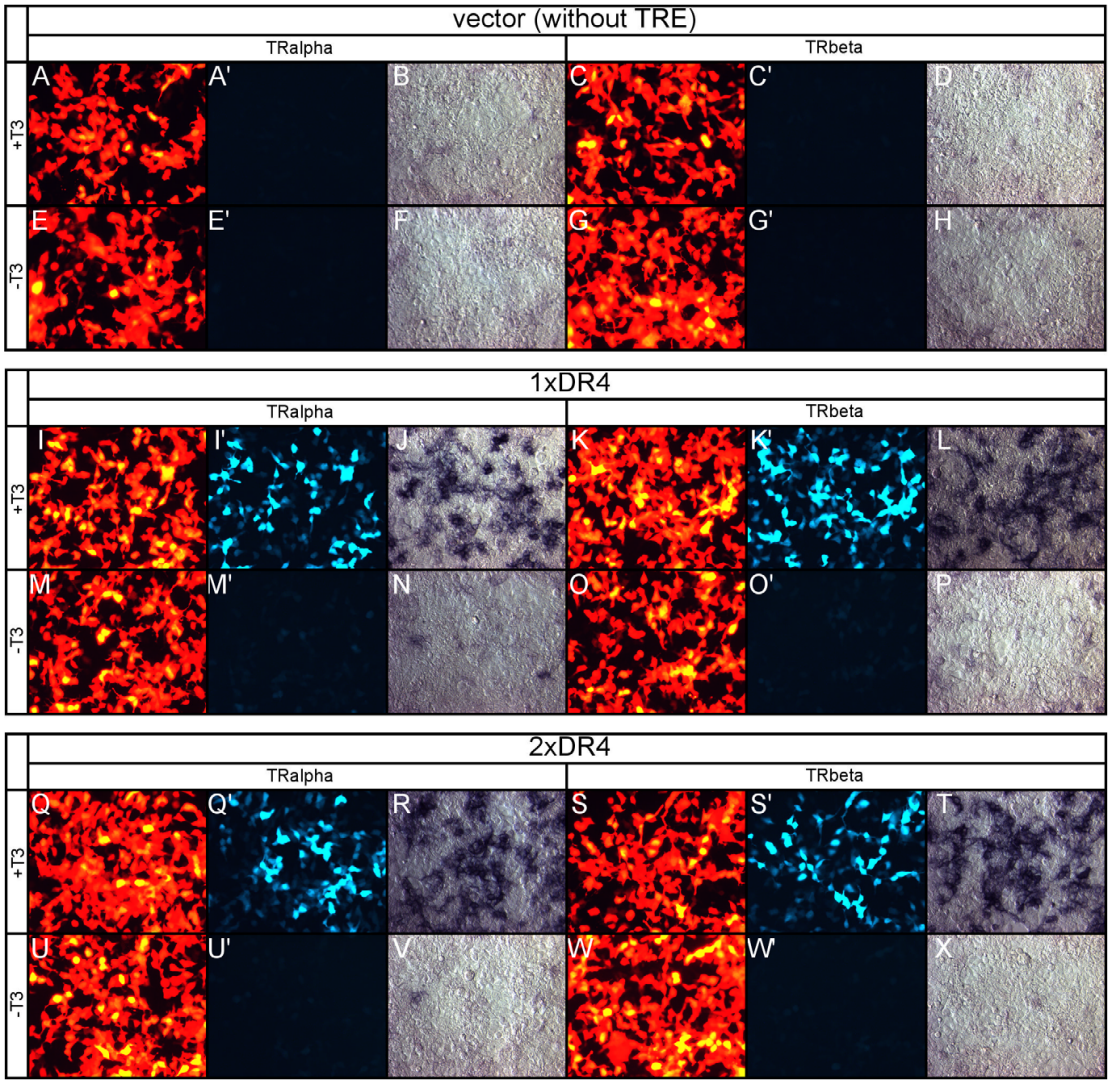
Reporter backbone, 1xDR4, and 2xDR4 tested in 293T cell-line. Each series of panels is labeled in the same order: A - RFP, A' - GFP, B - PLAP + either TRα or TRβ for the labeled TRE indicated ±T3. For each series, A and A' are the same field of view, whereas B is a different field of view. A–H. Empty vector (without TRE upstream of TATA) tested for T3 response via TRα or TRβ in cell culture. Empty vector, TRα (A–B, E–F) or TRβ (C–D, G–H), and a pβactin-RFP co-transfection marker were transfected into 293T cells in the presence of 100 nM T3 (A–H) or in the absence of exogenously added T3 (E–H). RFP fluorescence from co-electroporation plasmid with TRα +T3 (A), TRα -T3 (E), TRβ +T3 (C), and TRβ -T3 (G). GFP fluorescence from empty reporter with TRα +T3 (A'), TRα -T3 (E'), TRβ +T3 (C'), and TRβ -T3 (G'). Alkaline phosphatase activity from empty reporter with TRα +T3 (B), TRα -T3 (F), TRβ +T3 (D), and TRβ -T3 (H). I-P. 1xDR4 reporter tested for T3 responsiveness in cell culture. 1xDR4 + TRα±T3 (I–J, M–N). 1xDR4 + TRβ±T3(K–L, O–P). Q-X. 2xDR4 reporter tested for T3 responsiveness in cell culture. 2xDR4 + TRα±T3 (Q–R, U–V). 2xDR4 + TRβ±T3 (S–T, W–X).

To investigate the ability of each TRE to positively respond to T3 in the context of a minimal TATA, each construct was transfected into 293T cells and cultured ±T3. Each transfection included either a mouse (m) mTRα or rat (r) rTRβ expression vector, and a plasmid encoding the β-actin promoter driving RFP as a transfection control. After 24 hours, cell cultures were assayed for both RFP and GFP fluorescence, as well as alkaline phosphatase (AP) enzymatic activity. A control vector (Fig. 1B) with no TRE displayed no detectable GFP fluorescence (Fig. 2A', 2C', 2E', 2G') and a very low level of AP activity (Fig. 2B, 2D, 2F, 2H). However, transfection was successful as there was bright RFP fluorescence (Fig. 2A, 2C, 2E, 2G). In fact, the β-actin promoter drove RFP expression at such high levels that a few transfected cells appeared yellow in the RFP channel due to over-saturation (Fig. 2A, 2C, 2E, 2G). However, there was no bleed through from these over-saturated cells into the GFP channel (Fig. 2A', 2C', 2E', 2G'). No differences in these background signals were observed ±T3. Each TRE was qualitatively assayed for its ability to promote GFP and AP activity in response to T3 addition, or repress GFP and AP activity in the absence of exogenous T3, to verify that these previously validated T3-responsive TREs [22], [59]–[67] were still able to promote T3-dependent activity using only a minimal TATA.

#### 1xDR4 in cell culture

The optimized T3-responsive TRE, 1xDR4 (Fig. 1E), consists of two direct repeats separated by a 4 nucleotide spacer (DR+4), and is activated in a ligand-specific manner by T3 [22]. This reporter showed robust GFP fluorescence upon T3 addition when co-transfected with both TRα (Fig. 2I') or TRβ (Fig. 2K') eliciting a strong T3-induced response. However, in the absence of T3, no detectable GFP fluorescence was observed (Fig. 2M', 2O'). Likewise, strong AP activity was observed in the +T3 condition (Fig. 2J, 2L), whereas minimal AP activity was observed in the absence of T3 (Fig. 2N, 2P).

#### 2xDR4 in cell culture

The DR4 element was dimerized (Fig. 1F) to allow for investigation of whether a T3-sensitive activity could be further enhanced. The 2xDR4 displayed a robust T3 response mediated through both TRα (Fig. 2Q') and TRβ (Fig. 2S') in a similar fashion to what was observed with the 1xDR4 construct. Additionally, robust AP activity was induced by T3 (Fig. 2R, 2T). Similar to the 1xDR4, in the absence of T3, no GFP fluorescence and minimal AP activity were observed (Fig. 2U-X).

#### aTRE in cell culture

**Figure 3:**
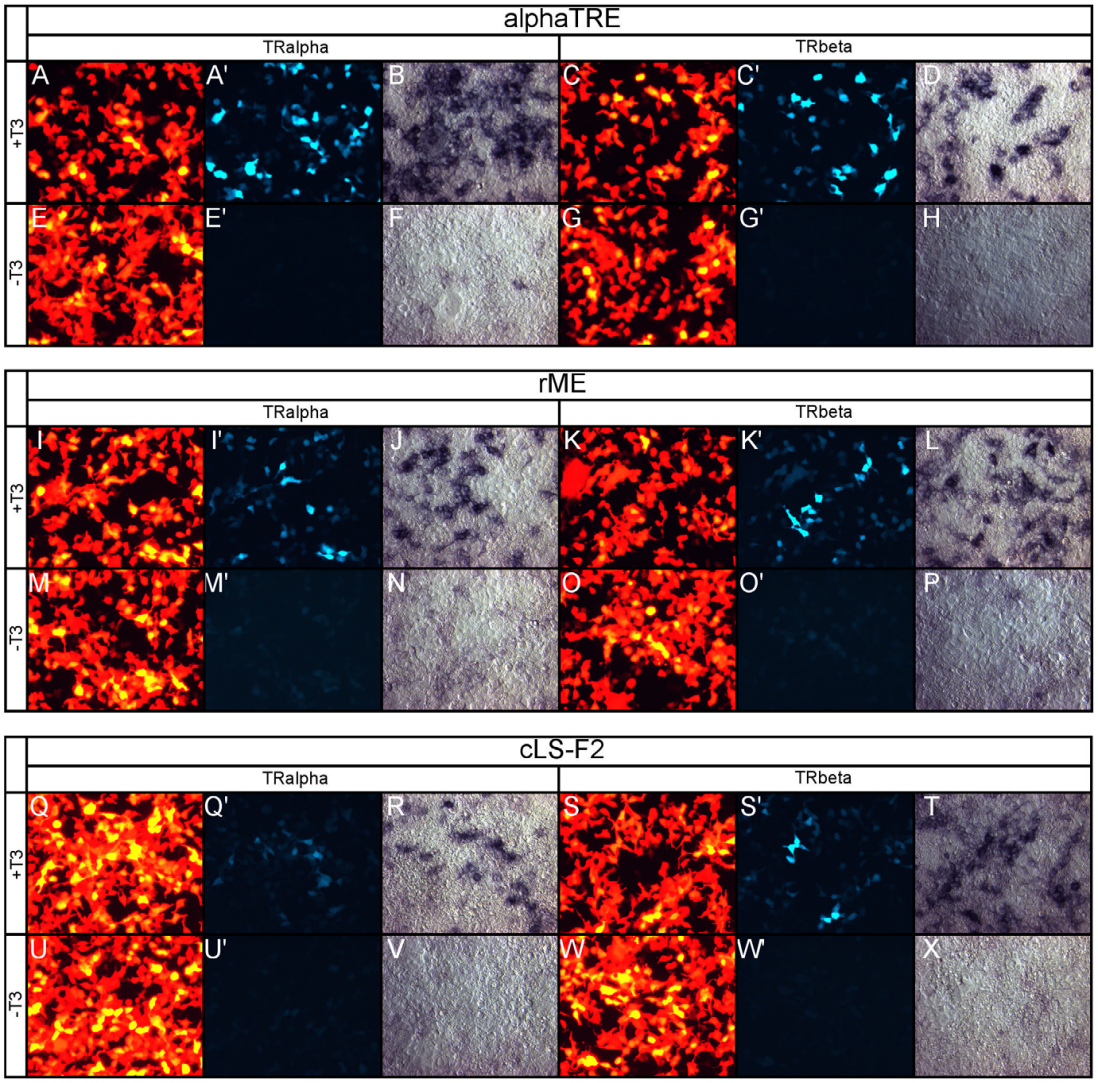
alphaTRE, rME, and cLS-F2 tested in 293T cell-line. Each series of panels is labeled in the same order: A - RFP, A' - GFP, B - PLAP + either TRα or TRβ for the labeled TRE indicated ±T3. For each series, A and A' are the same field of view, whereas B is a different field of view. A–H. aTRE tested for T3 response via TRα or TRβ in cell culture. aTRE + TRα±T3 (A–B, E–F). aTRE + TRβ±T3 (C–D, G–H). I–P. rME reporter tested for T3 responsiveness in cell culture. rME + TRα±T3 (I–J, M–N). rME + TRβ±T3 (K–L, O–P). Q-X. cLS-F2 reporter tested for T3 responsiveness in cell culture. cLS-F2 + TRα±T3 (Q–R, U–V). cLS-F2 + TRβ±T3 (S–T, W–X).

The DR+4 motif is present in other TREs, such as the compound αTRE (Fig. 1C), which consists of a 3^rd^ half site 19 bases 3' of a DR+4. This TRE has been shown to be preferentially activated by TRα [59]. The aTRE drove strong GFP fluorescence in the presence of either TRα or TRβ, and T3 (Fig. 3A', 3C'), with strong fluorescence and AP activity observed when TRα was transfected (Fig. 3B). Strong AP activity was also observed in the presence of TRβ (Fig. 3D), though in a smaller subset of cells. In the absence of T3, no GFP fluorescence and minimal AP activity were observed (Fig. 3E-H).

#### rME in cell culture

The rME (Fig. 1D) consists of 3 half sites in direct repeats separated by 3 and 4 bases [60], [61]. The rME drives GFP expression in a T3-dependent manner, although only in a subset of transfected cells (Fig. 3I-I', 3K-K'). AP detection showed a strong T3-induced response through both TRα (Fig. 3J) and TRβ (Fig. 3L). However, in the absence of T3 no GFP fluorescence and minimal AP activity were observed (Fig. 3M-P).

#### cLS-F2 in cell culture

The chicken lysozyme silencer F2 (cLS-F2) element (Fig. 1H) contains an everted palindrome and has been shown to have high affinity for T3-bound TR homodimers, leading to high T3-induced activity [62], [63]. However, in the 293T transfection assay, cLS-F2 only drove weak GFP fluorescence in a small subset of cells in the presence of TRα +T3 (Fig. 3Q'). Sensitive AP detection revealed additional TRα mediated T3-induced reporter activity in a larger subset of transfected cells (Fig. 3R) than was detected by the GFP fluorescence. TRβ +T3 also drove GFP expression via the cLS-F2 element (Fig. 3S'). AP staining also revealed that TRβ activated cLS-F2 in a larger subset of cells (Fig. 3T) than TRα. In the absence of T3, no GFP fluorescence and minimal AP activity were observed (Fig. 3U-X).

#### rGH-PAL and rGH-ABC in cell culture

The T3-sensitive rat growth hormone promoter has yielded two different synthetic T3-responsive TRE permutations. The rGH-ABC (Fig. 1I) element has three half sites (denoted A, B, and C) spaced as a DR+4 (between A and B) and an imperfect palindrome between B and C separated by a single base [64], [65]. A mutation of C to A was made in the C element so as to create an optimal AGGTCA half site [64]. The rGH-PAL TRE (Fig. 1G) is the C half site from the ABC TRE arranged as a palindrome [64], [66]. In transfected 293T cells, rGH-PAL drove very weak GFP fluorescence in the presence of TR and T3 (Fig. S1A', C') in a subset of transfected cells. However, with the more sensitive AP enzymatic assay, a clear T3-induced activation was observed (Fig. S1B, D) in a subset of transfected cells. In the absence of T3, no GFP fluorescence and minimal AP activity were observed with TRα (Fig. S1E-F) or TRβ (Fig. S1H). However, the larger rGH-ABC (Fig. 1I), which contains a DR4 element, showed no GFP fluorescence in the presence of T3 (Fig. S1I', S1K') and minimal AP activity (Fig. S1J, S1L). Importantly, there was no detectable difference in either GFP fluorescence or AP activity between ±T3 (Fig. S1I-P).

#### T3-independent reporters co-localize with co-transfection marker

Examination of the expression of RFP from the control plasmid, pβactin-RFP, showed that a much greater number of cells were transfected than were labeled by the TRE constructs. This could be due to poor co-transfection rates and/or differences in sensitivity of the RFP vs. the GFP and AP assays. To investigate these possibilities, a plasmid with the broadly active CAG promoter driving GFP was co-transfected with pβactin-RFP [68], [69]. Nearly 100% of RFP+ cells were GFP+ regardless of hormone addition (Fig. S1Q-Q', S-S'). Likewise, pCAG-AP labeled all transfected cells and AP labeling was unchanged by T3 addition (Fig. S1R, T).

To summarize, six of the seven previously identified TREs tested, when cloned upstream of a minimal TATA element, were able to drive both GFP and PLAP reporter gene expression in a T3-dependent manner in a 293T cell-line.

### The use of an IRES sequence to express the PLAP reporter does not influence the cell-type specific expression of AP

While AP was a sensitive and easy method to detect activity of the reporters, one potential concern was that its expression in the retina was affected in a cell-type specific manner by its position downstream of an IRES sequence. To determine if this was the case, the broadly expressed CAG promoter [69] was cloned upstream of the TATA box in the Stagia3 vector and electroporated into chicken retinas for 2 days. Retinal sections were probed for AP using a monoclonal antibody to AP and this signal was compared to endogenous GFP driven off of the same promoter in the same cells. If the IRES affected expression of AP in a cell-type specific manner, then one would expect to see varying ratios of GFP to AP in a cell-type specific manner. While the signal from the AP antibody was not as strong as GFP fluorescence and localized to a different cellular compartment, there was a very good qualitative correspondence between the two signals (Fig. S2A-C). Importantly, no strongly GFP-positive cells appeared to be AP negative. The specificity of the PLAP antibody was observed by comparing electroporated patches to non-electroporated patches of the same retina (Fig. S2C). To control for non-specific binding of the Cy3 secondary antibody or bleed through from the green (GFP) to red (anti-AP) channel, the AP antibody was left out, and, as expected, no signal was detected in the red channel (Fig. S2D-F).

#### Knock-down of TRα and TRβ using shRNA in the chick

To determine if TRα and/or TRβ mediated TRE reporter activity in the assay of TREs in chick retinal organ cultures, multiple shRNA constructs were made directed against all isoforms of both receptors. The avian pβactin-cU6-shRNA vector was used to express the shRNAs [70]. This plasmid encodes a β-actin promoter driving RFP, followed by the chick U6 promoter driving a short hairpin cassette, such that RFP marks a cell expressing the shRNA. Additionally, a corresponding "sensor" plasmid consisting of the CAG promoter driving the TRα or TRβ target sequence, followed by an IRES-GFP, was also engineered [68]. Each sensor and corresponding hairpin construct were co-transfected into 293T cells and evaluated for knock-down of the GFP from the sensor (data not shown). The same strategy was employed to create a GAPDH hairpin, to serve as a control for a successful knock-down event as well as for the introduction of a shRNA. The optimal hairpin for each TR or GAPDH was chosen for the *in vitro* electroporation (IVEP) experiments.

### Experimental strategy for detecting TRE activity in a developing CNS tissue

**Figure 4:**
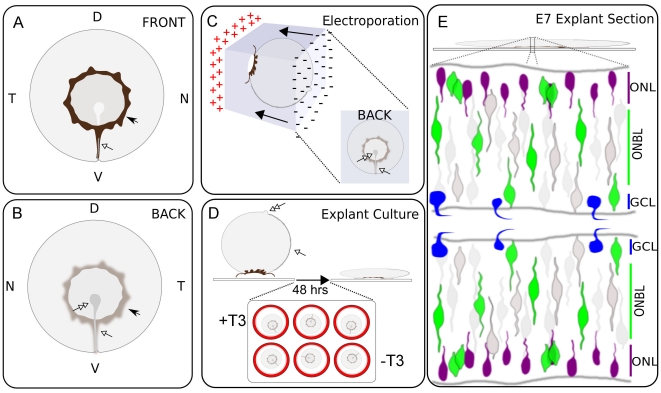
Retinal schematics and experimental strategy. A. Front-facing view of dissected retina from right eyeball. Abbreviations are as follows: Dorsal (D), Ventral (V), Temporal (T), and Nasal (N). Solid arrow head marks residual retinal pigmented epithelium (RPE) left around the lens to aid in manipulation of the retina. Open arrow head marks the ventral fissure landmark. B. Back-facing view of dissected right retina. Solid arrow head marks residual RPE located on the front-side that can visualized through the semi-translucent retina. Open arrow head marks the ventral fissure and the open double arrow head marks termination of the ventral fissure at the optic nerve head. C–D. Ex vivo experimental strategy. Dissected retinae were uniformly orientated in a cocktail of TRE reporter and pβactin-RFP plasmid and in vitro electroporated such that the majority of the plasmid was delivered to the posterior surface of each retina (C). The lens was then removed and each retina placed posterior surface facing up on a filter floating in media (D). Over 48 hrs, the retinae compressed under their own weight such that whole-mount images resemble the schematic in (B). E. Schematic of retinal explant section during early chick development. Abbreviations are as follows: outer nuclear layer (ONL) and ganglion cell layer (GCL). Early in development there are three easily defined nuclear layers, based on cellular morphologies: developing ONL, GCL, and progenitor zone. Generally, cycling progenitors undergo S-phase of the cell cycle near the GCL and M-phase near the ONL. Dio2 and TRβ2 mRNA expression restricted to the ONL. Dio3 mRNA expression restricted to the progenitor zone. TRα mRNA expression is hazy and spans both the progenitor zone as well as the ONL.

Each TRE reporter was tested for its activity in the chick retina. The chick retina was chosen as we had previously found dynamic expression of TR signaling components [41] and it lends itself to the detection of patterns due to its large size. To this end, chick retinae were harvested at embryonic day 5 (E5), and the retinal pigmented epithelium (RPE) was carefully removed, leaving a small remnant around the lens and ventral fissure for ease in manipulation of the retina (Fig. 4A-B). Retinae were then electroporated *in vitro* with each reporter and RFP/GAPDH-shRNA, using the ventral fissure and optic nerve head as landmarks. They were then cultured *ex vivo* for 48 hours in varying amounts of T3 (Fig. 4C-D). Reporter AP activity was assayed after 48 hours, at the equivalent of E7. At E7, a dynamic wave of Dio2 and Dio3 expression sweeps through the avian retina to create what might be a wave of TH activation in ONL and/or progenitor cells, with subsequent inactivation due to Dio3 expression in progenitor cells (Fig. 4E) [41]. We reasoned this would be an ideal point in development to observe TH signaling.

### Control vector has minimal activity in the retina

Consistent with the results observed from transfection of 293T cells (Fig. 2A-H), the control reporter vector with no TRE upstream of the TATA showed minimal AP activity ±T3 in the chick retina (Fig. S3A-D). *In vitro* electroporation robustly delivered plasmid to the retina as assayed by the co-electroporation marker RFP (Fig. S3A'-D'; red channel shows RFP and marks GAPDH-shRNA delivery). Cryosectioning revealed high RFP fluorescence and virtually zero AP activity, with only an occasional apical cell faintly marked in the developing ONL in a non-T3 sensitive manner (Fig. S3E-F'). Electroporation of either TRα or TRβ-shRNA had no effect on the occasional AP expression from the empty vector in either ±T3 (Fig. S3G-H').

### aTRE drives PLAP expression in the developing retina in a T3-dependent manner

**Figure 5:**
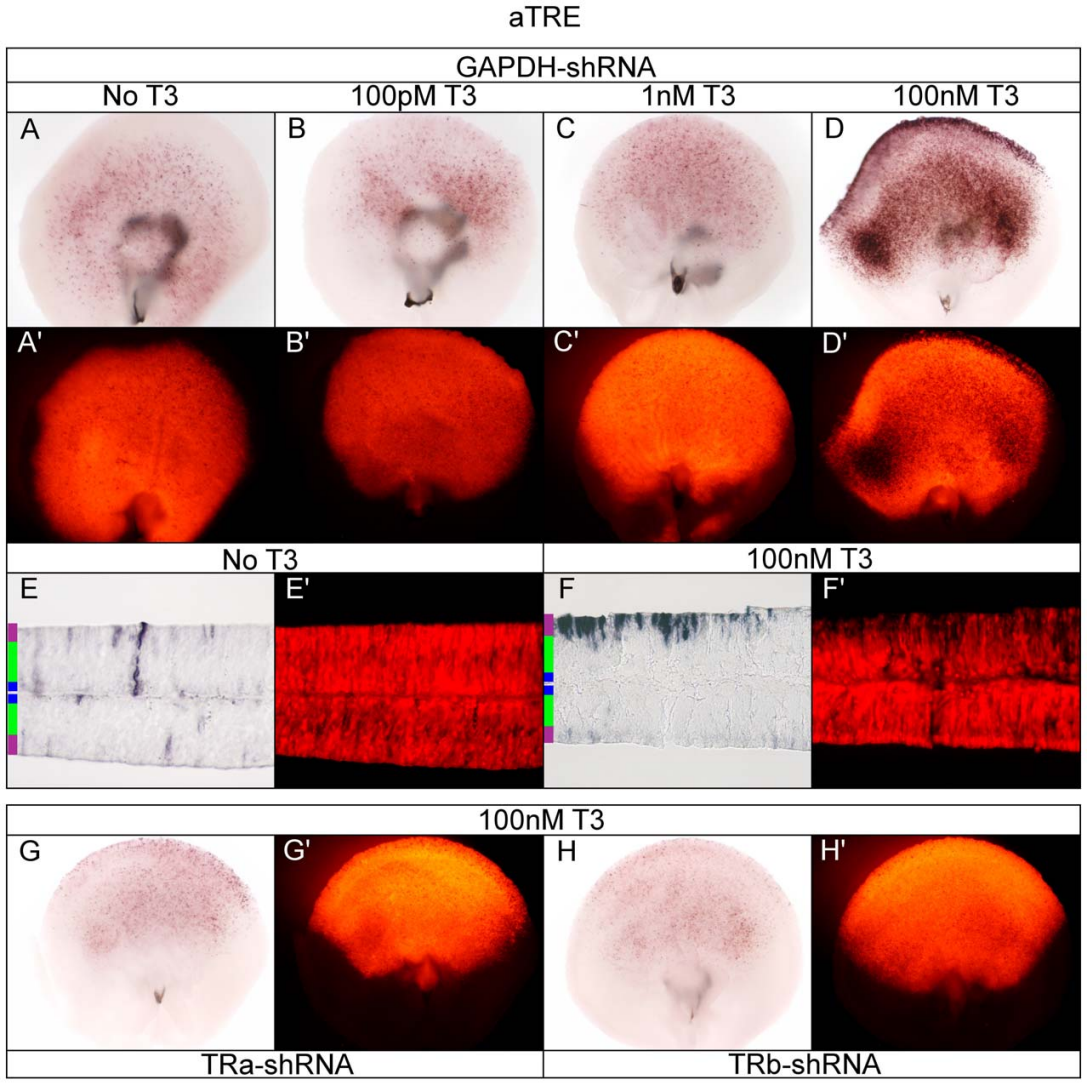
Assay of the aTRE reporter in the developing chick retina. A–D. Embryonic day 5 retinae were electroporated with aTRE + pβactin-RFP/GAPDH-shRNA in varying T3 concentrations. aTRE + GAPDH-shRNA -T3 (A, A'), 100 pM T3 (B, B'), 1 nM T3 (C, C'), and 100 nM T3 (D, D'). E–F. Cryosectioning of retina. 20 um cryosection of aTRE + GAPDH-shRNA -T3 (E, E') and 100 nM T3 (F, F'). G–H. TRα and TRβ shRNA in 100 nM T3. aTRE in 100 nM T3 + TRα-shRNA (G, G') or TRβ-shRNA (H, H').

TRα is expressed fairly ubiquitously, and at low levels, in the developing chick retina. This includes cells with a progenitor morphology, which also express the TH-degrading enzyme, Dio3 [41]. These observations raise the question as to whether retinal progenitor cells can respond to a TH signal, if cultured in enough T3 to overcome Dio3 activity. To address this question, the aTRE, which was activated in cell culture by T3+TRα, was electroporated into E5 retina. Consistent with the previous literature, as well as the results observed from transfection of 293T cells (Fig. 3A-H), the aTRE was strongly activated in the retina in 100 nM T3 (Fig. 5D).

Cryosectioning revealed that AP-positive cells localized to the apical side of the developing retina, with a morphology consistent with that of a newly postmitotic PR cell arriving at its final position (Fig. 5F). The AP pattern is similar to that of TRβ expression at this point in development [41]. Consistent with the results observed from transfection of 293T cells (Fig. 3A-A', C-C'), AP labeling appeared only in a subset of electroporated cells marked with RFP (Fig. 5F-F'). However, unlike the cell culture data, the aTRE showed above basal levels of AP activity in no T3 or low T3 concentrations (Fig. 5A-C). However, without T3 added to the system, activation was low. In no/low T3 concentrations, the AP activity was localized to cells whose processes spanned the radial dimension, with a morphology consistent with that of a progenitor cell (Fig. 5E). Additionally, low levels of AP were detectable in the developing ONL. Knock-down of TRα or TRβ in 100 nM T3 resulted in a substantial reduction in aTRE activation (Fig. 5G, 5H) as compared to the GAPDH-shRNA control (Fig. 5D). This indicated that aTRE activity in 100 nM T3 can be mediated through either TRα and TRβ.

### Dorsalized TR-dependent rME activity in developing ONL

**Figure 6:**
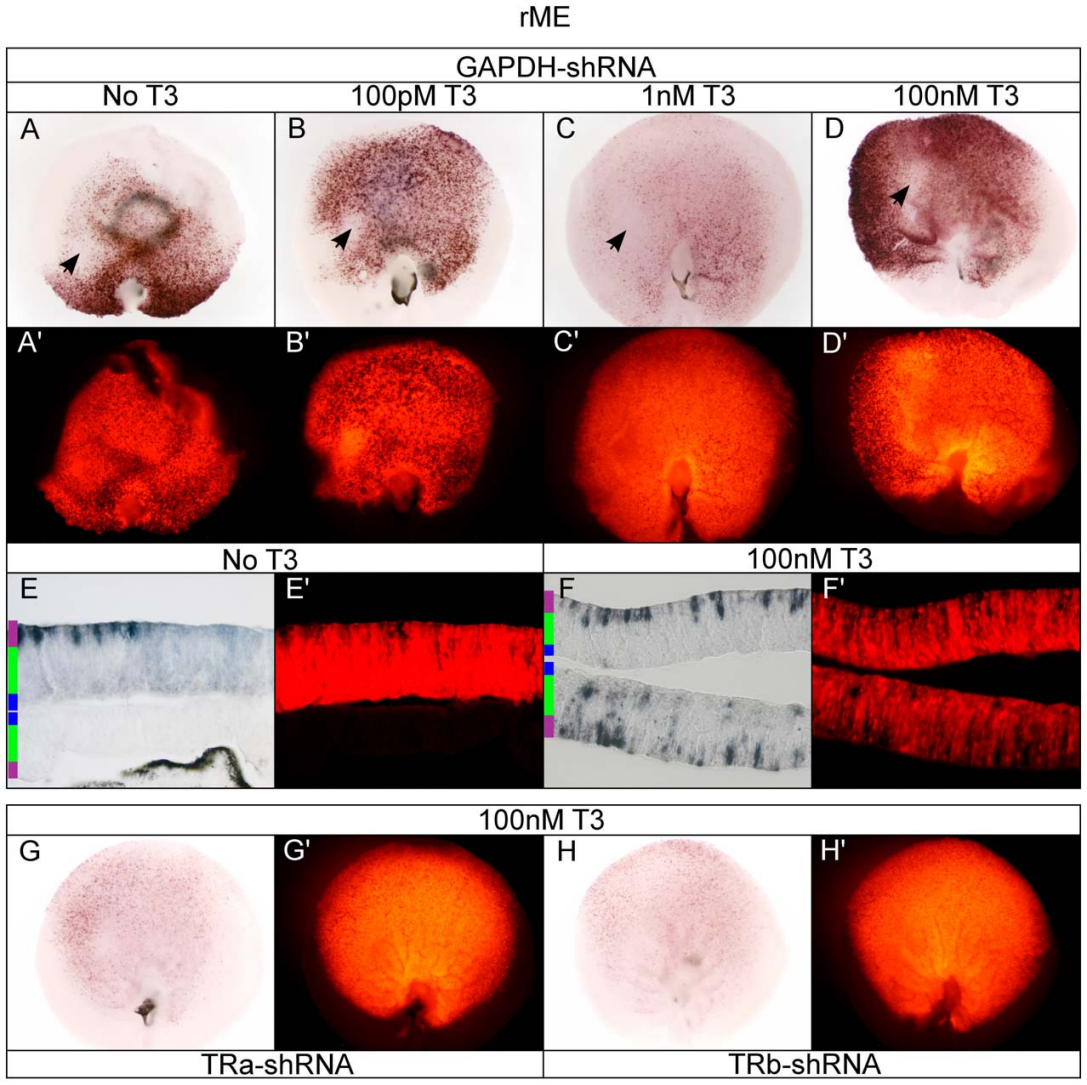
Assay of the rME reporter in the developing chick retina. A–D. rME + pβactin-RFP/GAPDH-shRNA in varying T3 concentrations. rME + GAPDH-shRNA -T3 (A, A'), 100 pM T3 (B, B'), 1 nM T3 (C, C'), and 100 nM T3 (D, D'). E–F. Cryosectioning of retina. 20 um cryosection of rME + GAPDH-shRNA -T3 (E, E') and 100 nM T3 (F, F'). Closed arrow heads mark AP-negative patch. G–H. TRα and TRβ shRNA in 100 nM T3. rME in 100 nM T3 + TRα-shRNA (G, G') or TRβ-shRNA (H, H').

The rME reporter strongly activated in 100 nM T3 in the retina with the exception of a notable AP-negative patch in the central-nasal part of the retina, which was observed in all T3 conditions (Fig. 6A-D). The AP-negative patch was successfully electroporated as indicated by the robust RFP signal (Fig. 6A'-D'). The AP-negative patch was detected both at lower and higher levels of exogenous T3 (Fig. 6B-D). There was a reduction in AP activity in 1 nM T3 (Fig. 6C) as compared to either 100 pM (Fig.6B) or 100 nM T3 (Fig. 6D) which might reflect competition by other factors for the multiple direct repeats present in the rME (Fig. 1D). However, the AP-negative patch appeared unaffected. Without exogenous T3 added to the system, a striking ventralized pattern emerged that resembled both early Dio2 RNA expression, as well as the later expression of the rod gene, rhodopsin [71] (Fig. 6A-A'). The AP+ cells in the ventral retina were restricted to the developing ONL, with a decreasing number of AP+ ONL cells across the dorsal retina (Fig. 6E-E'). Notably, the AP-negative central/nasal patch was still observed in the absence of T3. In high T3, there was high rME AP activity in ONL cells, as well as additional activity in cells in the progenitor zone and a few in the ganglion cell layer (GCL) (Fig. 6F). The ventral pattern observed in the absence of exogenous T3 was TR-dependent, as TRα or TRβ knock-down resulted in loss of both dorsal and ventral rME activity in 100 nM T3 (Fig. 6G-H' compared to Fig. 6D-D').

### TR-independent dorsal and T3-dependent ventral 1xDR4 activity

**Figure 7:**
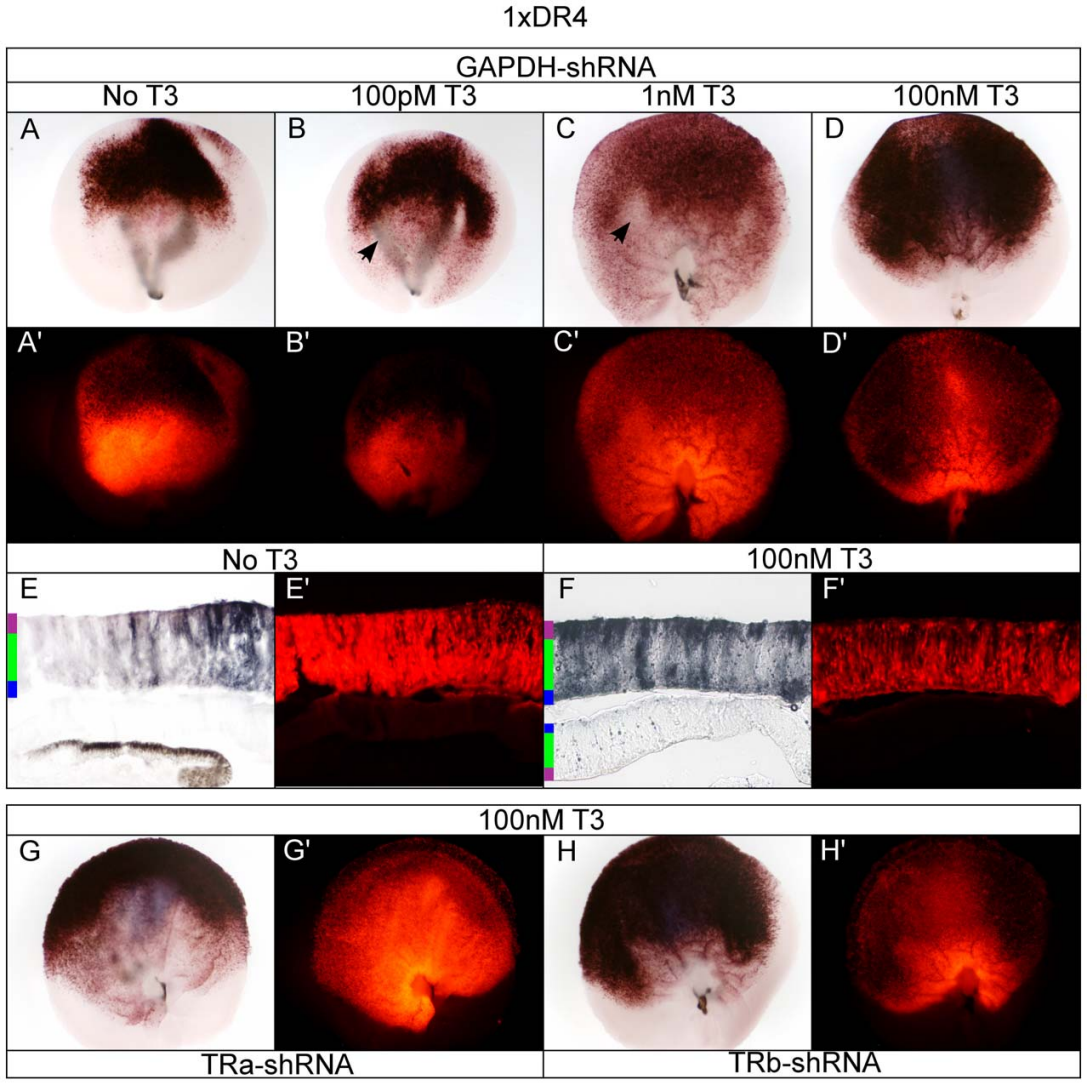
Assay of the 1xDR4 reporter in the developing chick retina. A–D. 1xDR4 + pβactin-RFP/GAPDH-shRNA in varying T3 concentrations. 1xDR4 + GAPDH-shRNA -T3 (A, A'), 100 pM T3 (B, B'), 1 nM T3 (C, C'), and 100 nM T3 (D, D'). Closed arrow heads mark AP-negative patch. E-F. Cryosectioning of retina. 20 um cryosection of 1xDR4 + GAPDH-shRNA -T3 (E, E') and 100 nM T3 (F, F'). G–H. TRα and TRβ shRNA in 100 nM T3. 1xDR4 in 100 nM T3 + TRα-shRNA (G, G') or TRβ-shRNA (H, H').

The DR+4 sequence is the canonical T3-response element. It showed a highly specific ligand response to T3 in cell culture, and was specific to T3 in that it did not show response to RA or VitD3 in previous studies [22]. In the developing chick retina, there was robust 1xDR4 directed AP activity in 100 nM T3 (Fig. 7D). Interestingly, in the absence of added T3, a pronounced dorsal activity was observed, along with a complete lack of ventral 1xDR4 activity (Fig. 7A). As the T3 concentration was increased, ventral 1xDR4 activation was observed (Fig. 7A-D). Total AP activity in 100 nM T3 (Fig. 7D') and dorsal AP activity without T3 (Fig. 7A') was at high enough levels that a substantial portion of the RFP fluorescence was quenched, even though all AP reactions were deveoped for the same amount of time. However, in 1 nM T3 dorsal 1xDR4 activity decreased as compared to 100 pM and 100 nM T3 conditions, possibly reflecting a competition for either 1xDR4 binding or heterodimer partners, resulting in less efficient reporter activation (Fig. 7C). An AP-negative patch was also observed in the central/nasal retina at 100 pM and 1 nM T3 concentrations, when ventral activation became apparent (Fig. 7B-C). At these intermediate T3 concentrations ventral activation was primarily observed in the ONL (data not shown). Strong dorsal AP activity in the absence of T3 localized to both the developing ONL as well to cells with processes spanning the radial dimension, consistent with the morphology of a progenitor cell, which faded in the ventral part of the retina (Fig. 7E). In 100 nM T3, AP activity was high in the ONL, as well as the GCL (Fig. 7F). Knock-down of TRα in high T3 resulted in loss of substantial AP activity in the central and ventral retina (and subsequent increase in the ability to visualize RFP fluorescence). Dorsal 1xDR4 directed AP activity appeared relatively unperturbed when TRα activity was knocked-down (Fig. 7G-G' compared to Fig. 7D-D'). TRβ knock-down resulted in no appreciable loss of AP activity nor gain in RFP fluorescence (Fig. 7H-H' compared to Fig. 7D-D'), indicating that TRβ's role in 1xDR4 activation in the dorsal retina may be minimal.

### Strong T3-dependent 2xDR4 activity in the developing ONL

**Figure 8:**
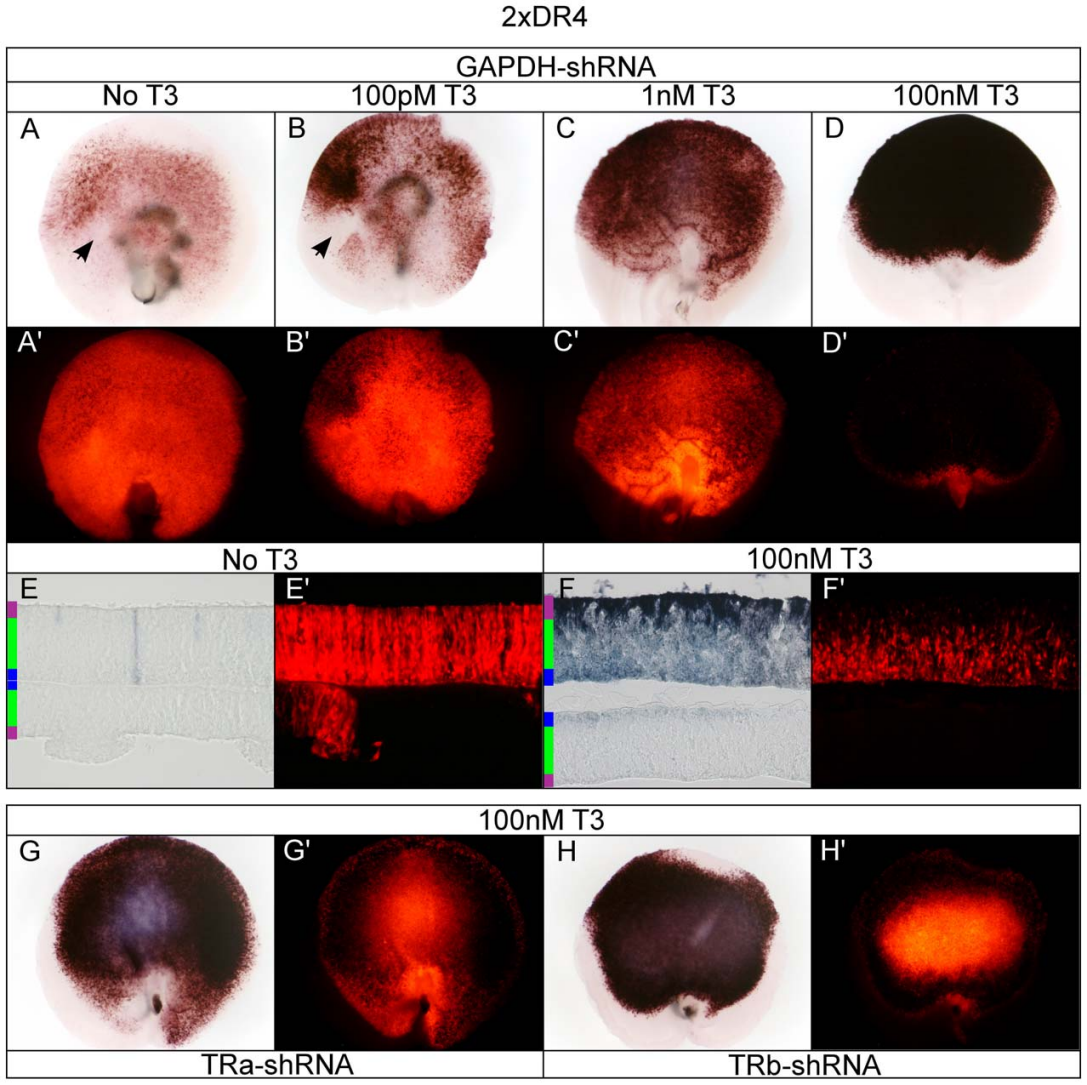
Assay of the 2xDR4 reporter in the developing chick retina. A–D. 2xDR4 + pβactin-RFP/GAPDH-shRNA in varying T3 concentrations. 2xDR4 + GAPDH-shRNA -T3 (A, A'), 100 pM T3 (B, B'), 1 nM T3 (C, C'), and 100 nM T3 (D, D'). Closed arrow heads mark AP-negative patch. E–F. Cryosectioning of retina. 20 um cryosection of 2xDR4 + GAPDH-shRNA -T3 (E, E') and 100 nM T3 (F, F'). G–H. TRα and TRβ shRNA in 100 nM T3. 2xDR4 in 100 nM T3 + TRα-shRNA (G, G') or TRβ-shRNA (H, H').

In contrast to the highly dorsalized pattern of the single DR+4 element in the absence of T3, adding a second DR4 element resulted in a significant reduction in dorsal activity and slight gain in ventral activity (Fig. 8A-A'). A small central/nasal AP-negative patch was observed in the absence of T3 as well as in 100 pM T3. On section, minimal AP staining was observed without T3 addition, and activity was restricted to an occasional cell in the ONL or ONBL (outer neuroblastic layer) (Fig. 8E-E'). Increasing T3 concentration resulted in an increase in both ventral and dorsal 2xDR4 AP activity (Fig. 8B-C) which was highest in the ONL (data not shown). High 100 nM T3 activated the 2xDR4 reporter so robustly that virtually all of the RFP fluorescence was quenched, even though all AP reactions were developed for the same amount of time (Fig. 8D-D'). In high T3, AP activity was high in the developing ONL although hazy AP activity, likely due to faintly labeled cells, was observed throughout the radial dimension of the retina (Fig. 8F-F'). No AP+ cell bodies were observed in the ONBL or the GCL. Like the 1xDR4, knock-down of TRα resulted in a partial loss of AP activity and gain of RFP fluorescence in the central retina, though the periphery retained high AP activity (Fig. 8G-G' compared to 8D-D'). TRβ knock-down resulted in a reduction of AP activity in the central retina, as inferred from the substantial increase in corresponding RFP fluorescence (Fig. 8H-H' compared to 8D-D'), indicating that TRβ may be playing a role in activating the 2xDR4 in high T3 in this region.

### Knock-down of TRα and TRβ: Effect on 1xDR4 and 2xDR4

**Figure 9:**
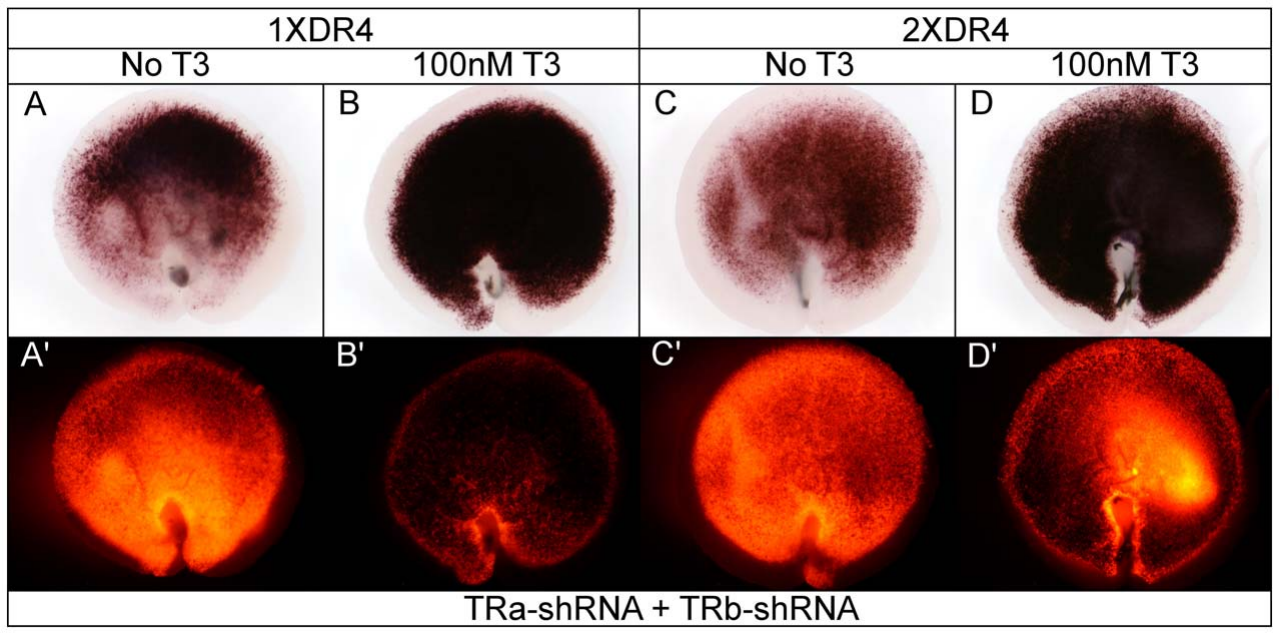
Assay of DR4 reporters with TRα/TRβ double knock-down. A–B. 1xDR4 + TRα/TRβ-shRNA without T3 (A, A') or +100 nM T3 (B, B'). C–D. 2xDR4 + TRα/TRβ-shRNA without T3 (C, C') or +100 nM T3 (D, D').

To further investigate the roles of TRα and TRβ in driving DR4 reporter activity, double TR shRNA was analyzed ±T3. Knock-down of TRα and TRβ together in the absence of T3 resulted in de-repression of 1xDR4 activity ventrally, but did not perturb the high dorsal activity (Fig. 9A-A' compared to Fig. 7A-A'). However, TRα/TRβ-shRNA in the absence of T3 resulted in an equivalent dorsal and ventral de-repression of 2xDR4 (Fig. 9C-C' compared to Fig. 8A-A') but the dorsal 2xDR4 activity was lower than that of dorsal 1xDR4 activity (Fig. 9A compared to 9C). Unlike the single TRα-shRNA, double knock-down resulted in increased 1xDR4 activity in the central retina in +T3 (Fig. 9B-B' compared to Fig. 7G-G'). Conversely, double TR knock-down resulted in loss of 2xDR4 central activity in high T3 (Fig. 9D-D') which was similar to the activity observed with single shRNA to either TRα or TRβ (Fig. 8G-H').

### TRs repress rGH-PAL in a T3-dependent manner

**Figure 10:**
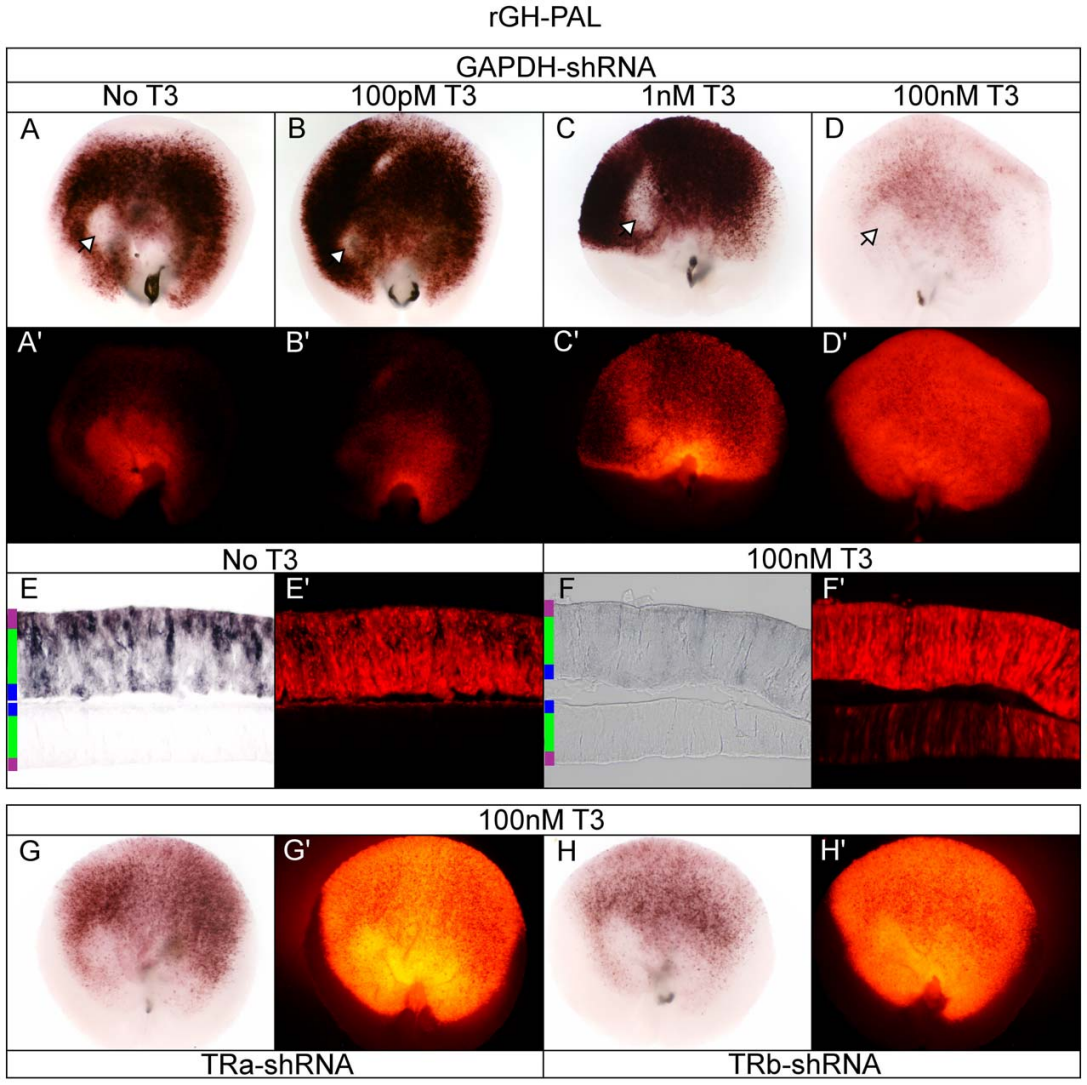
Assay of the rGH-PAL reporter in the developing chick retina. A–D. rGH-PAL + pβactin-RFP/GAPDH-shRNA in varying T3 concentrations. rGH-PAL + GAPDH-shRNA -T3 (A, A'), 100 pM T3 (B, B'), 1 nM T3 (C, C'), and 100 nM T3 (D, D'). Open arrow heads mark AP-negative patch. E–F. Cryosectioning of retina. 20 um cryosection of rGH-PAL + GAPDH-shRNA -T3 (E, E') and 100 nM T3 (F, F'). G–H. TRα and TRβ shRNA in 100 nM T3. rGH-PAL in 100 nM T3 + TRα-shRNA (G, G') or TRβ-shRNA (H, H').

**Figure 11:**
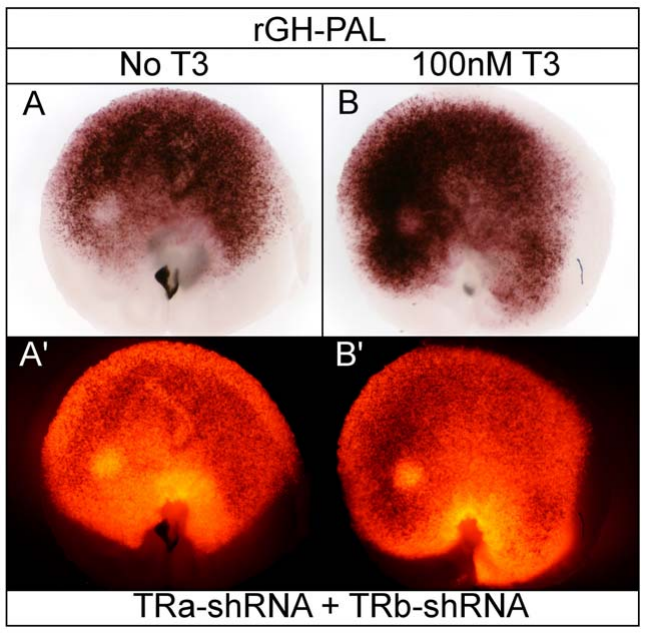
Assay of rGH-PAL with TRα/TRβ double knock-down. A. rGH-PAL + TRα/TRβ-shRNA in the absence of added T3. B. rGH-PAL + TRα/TRβ-shRNA cultured in 100 nM T3.

In 293T cells, rGH-PAL drove weak GFP fluorescence and moderate AP activity in a T3-dependent manner. In the absence of T3 the sensitive rGH-PAL PLAP reporter gene was silent (Fig. S1A-D'). In the retina however, intense rGH-PAL AP activity was observed in the absence of T3, with the exception of a central/nasal AP-negative patch, as well as the optic nerve head (Fig. 10A-A'). Both the intense AP activity as well as the central/nasal AP-negative patch were unchanged by increasing T3 concentrations up to 1 nM (Fig. 10B-C'). However, in contrast to cell culture data, the rGH-PAL activity was substantially repressed rather than activated in 100 nM T3 concentrations (Fig. 10D-D'). Intriguingly, T3 addition had no effect on the AP-negative patch. Knock-down of TRα resulted in a substantial relief of T3-induced repression, although the shRNA didn't perturb the central/nasal AP-negative patch (Fig. 10G-G' compared to 10D-D'). TRβ knock-down also resulted in relief of T3-induced rGH-PAL repression, though to a slightly lesser extent than TRα knock-down (Fig. 10H-H' compared to 10D-D'). On section, AP+ cells in the absence of T3 loosely localized to the ONL, with some AP+ cell bodies found in the ONBL as well as a few in the GCL (Fig. 10E-E'). In contrast, in 100 nM T3, AP activity was virtually absent on section with only a few hazy outlines of cells in the ONBL and ONL (Fig. 10F-F'). In light of the unexpected rGH-PAL T3-dependent repression, the double TR-shRNA condition was analyzed ±T3 to investigate if the repression was additive. Double knock-down of TRα and TRβ in the retina in 100 nM T3 resulted in additive relief of repression (Fig. 11B-B' compared to Fig. 10D-D', 10G-H'). Equivalent AP activity was observed upon double TR-shRNA in the absence of T3 indicating the rGH-PAL activity was both TR and T3-independent (Fig. 11A-A').

### cLS-F2 is minimally active through TRα and TRβ, but is not T3-sensitive in the retina

**Figure 12:**
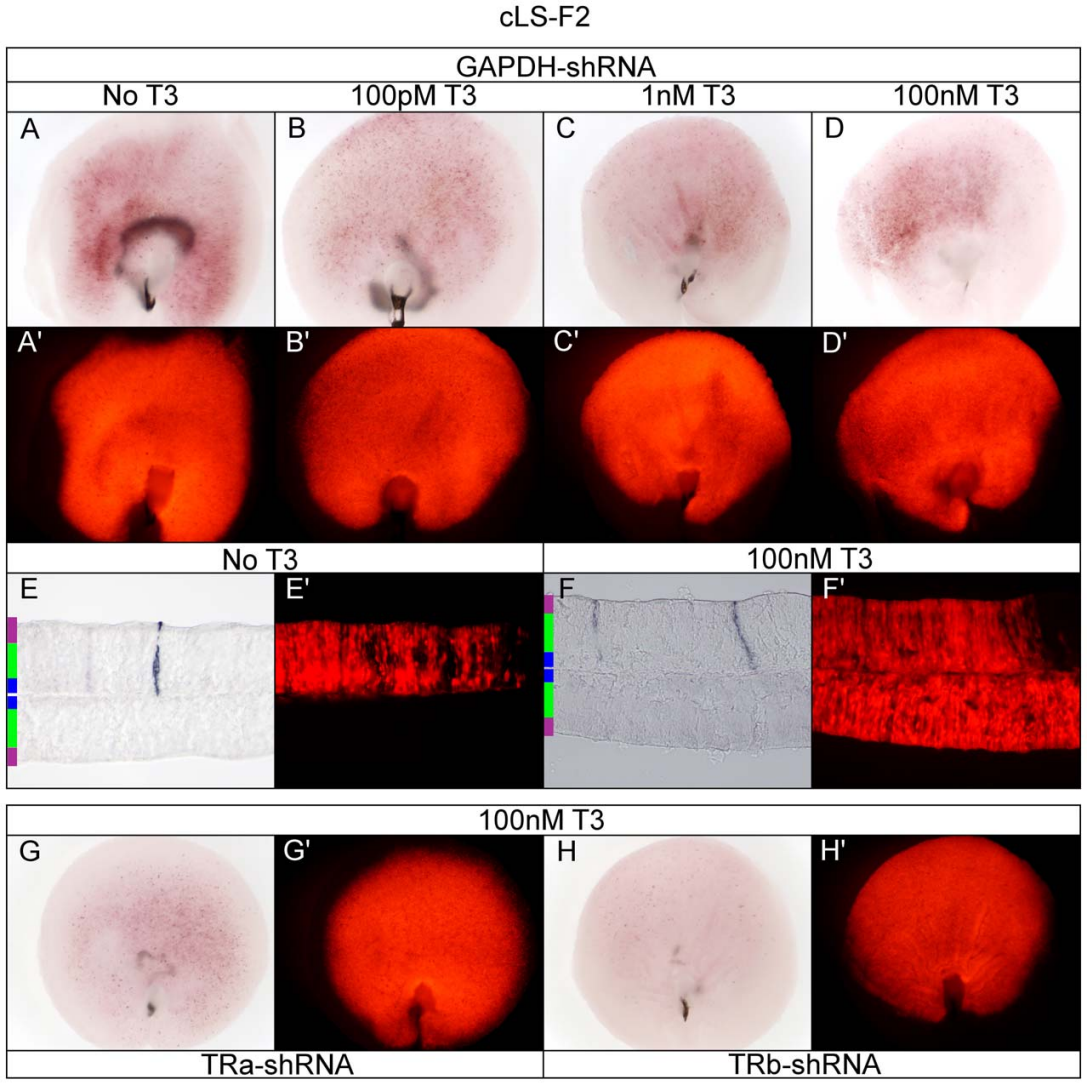
Assay of the cLS-F2 reporter in the developing chick retina. A–D. cLS-F2 + pβactin-RFP/GAPDH-shRNA in varying T3 concentrations. cLS-F2 + GAPDH-shRNA -T3 (A, A'), 100 pM T3 (B, B'), 1 nM T3 (C, C'), and 100 nM T3 (D, D'). E–F. Cryosectioning of retina. 20 um cryosection of cLS-F2 + GAPDH-shRNA -T3 (E, E') and 100 nM T3 (F, F'). G–H. TRα and TRβ shRNA in 100 nM T3. cLS-F2 in 100 nM T3 + TRα-shRNA (G, G') or TRβ-shRNA (H, H').

In contrast to the results observed from transfection of 293T cells, where cLS-F2 was T3-responsive (Fig. 3Q-T), the cLS-F2 was not activated in a T3-dependent manner in the retina, although low AP activity for the cLS-F2 TRE was above control vector background (Fig. 12D-D'). The same above-basal level of activity was observed in the absence of T3 (Fig. 12A-A') as well as intermediate concentrations of T3 (Fig. 12B-C'). Interestingly, while there was only minimal AP labeling of cells in either ±T3 condition, all AP+ cells shared a progenitor-like morphology and localized to the ONBL (Fig. 12E-F'). This was unlike the occasional hazy apical ONL cells that were labeled by the control vector (Fig. S3E). Nevertheless, knock-down of TRα (Fig. 12G-G' compare to 12D-D') or TRβ (Fig. 12H-H' compare to 12D-D') resulted in reduction in the subtle above-basal AP levels to that of vector background indicating that the slight T3-independent activity that was observed was due in part to TRα and TRβ.

### Minimal rGH-ABC activity is TR-independent

Unlike the cLS-F2, the rGH-ABC TRE showed no T3-induced activity in the 293T cell culture assay (Fig. S1I-P). In the retina, the rGH-ABC showed slightly above background levels of AP activity in a non-T3-sensitive fashion (Fig. S4A-D'). However, the few cells that were labeled in both low and high T3 concentrations shared a progenitor-like morphology (Fig. S4E-F') unlike the cells labeled by control vector (Fig. S3E). Knock-down of either TRα or TRβ did not perturb the subtle AP pattern, indicating that both TRs have minimal roles in the basal rGH-ABC activity that was observed (Fig. S4G-H' compare to S3D-D').

## Discussion

### A minimal TATA-box combined with a minimal TRE allow for analysis of *in vivo* reporter activity

Appropriate levels of TH are absolutely critical for correct brain development through molecular mechanisms that aren't well understood [1]. Here we examine the ability of previously validated minimal TREs to drive reporter expression in a TR and T3-sensitive manner in a complex developing CNS tissue, the retina. Our approach differed from previously published studies in several ways. First, a 15bp minimal TATA element was used rather than the full-length TK or SV40 promoters, in order to lessen the amount of minimal promoter activity. Both TK and SV40 minimal promoters confer basal activity in the absence of any upstream TRE, which makes them sufficient for *in vitro* luciferase and CAT assays, but less ideal for analyzing TRE activity in tissue where the assay is not as quantitative. In this case, differentiating between SV40 activity and TRE activation would have been difficult, as the SV40 promoter alone is sufficient to drive the GFP-IRES-PLAP cassette in the retina in the absence of an upstream TRE (Emerson and Cepko, *unpublished*). By using a TATA-box with low intrinsic activity on its own, any *in vivo* reporter activity could be specifically attributed to receptors and co-factors interacting with the TRE. Indeed, the Stagia3 vector drove minimal expression of the GFP-IRES-PLAP reporter cassette with no detectable GFP fluorescence and barely detectable PLAP staining in both cell culture as well as in the retina. When the broadly active CAG promoter was cloned in front of the minimal TATA box, both GFP and PLAP showed broad expression as well as co-localization, indicating that the IRES element was not skewing expression of the PLAP reporter, by reporting in only a subset of electroporated cells (Fig. S2). AP enzymatic activity is an extremely sensitive read-out and the lack of AP activity in both cell culture as well as in the retina is evidence of the lack of intrinsic activity of the minimal TATA alone.

### Cell culture TRE activation versus de-repression

Six of the seven TREs tested using the minimal TATA-box showed various levels of T3-dependent activation of both reporter genes in cell culture. The rGH-PAL and cLS-F2 TREs showed only weak T3-activation. This was surprising in light of previous reports demonstrating robust T3-response using the TK promoter. Interestingly, the rGH-ABC did not drive T3-dependent reporter activity in cell culture, even though the sequence contains a DR+4 element. As the rGH-ABC element has previously been shown to be T3-sensitive in front of the TK promoter, lack of T3-dependent activation in our system may suggest that the rGH-ABC sequence is a more efficient repressor in the absence of T3, rather than an activator – something that our system was not designed to test. Similarly, the cLS-F2 element which had previously been demonstrated to have robust T3-sensitive activity when combined with the TK promoter, only showed mild T3-activation when combined with the minimal TATA. This could suggest that the sequence may be more efficient at T3-mediated de-repression rather than T3-dependent activation. If cLS-F2 has more potent binding of TRs and co-repressors in the absence of T3, but is only mildly successful at recruiting TR and co-activators in the presence of T3, then this may explain why the cLS-F2 showed mild T3-dependent activation in cell culture, but no appreciable T3-dependent activation in the retina. Another possibility is that the rGH-PAL, rGH-ABC, and cLS-F2 all require additional TRE sequence, present in the complete TK promoter, to enhance their T3 responsiveness [58]. Even so, the negative *in vivo* results for both cLS-F2 and rGH-ABC further indicate the specificity of these plasmid reporters, in that simply having a sequence upstream of the TATA is not sufficient to drive reporter activation in the retina.

### rGH-PAL displays TR and T3-independent activation

In the canonical model of T3-mediated activation, TRs are thought to remain bound to response elements in the nucleus in the absence of T3, and to repress transcription [72]–[75]. Following transfection of 293T cells, the rGH-PAL displayed a mild T3-dependent induction, with no reporter activation in the absence of exogenous hormone when TRα or TRβ were co-transfected. This reporter showed very different behavior in the retina. In the absence of exogenous T3, the rGH-PAL displayed robust activity which was substantially repressed in high T3 concentrations, through TRα and TRβ. The rGH-PAL T3-dependent repression was partially relieved in high T3 with knock-down of either TRα and TRβ. Double knock-down of TRα and TRβ in the retina in 100 nM T3 resulted in additive relief of repression and the high reporter activity was equivalent to double receptor knock-down in the absence of T3 (Fig. 11). These results indicate that the rGH-PAL activity can be both TR and T3-independent. Together, these data would suggest that in the complex milieu of the retina, TRs may not remain bound to the rGH-PAL element, and that in the absence of high T3 a different factor(s) binds the sequence and recruits the appropriate co-activator components to drive reporter activity. The low level of reporter activity in 100 nM T3 (Fig. 10D) is consistent with the low level of activation observed in cell culture (Fig. S1A', 4C').

In low T3 concentrations, the rGH-PAL displayed strongest activity in cells with a progenitor-like morphology, though a subset of ONL cells may also have been labeled (Fig. 10E). Previous studies have suggested that TH may induce rat retinal progenitor cells to exit the cell cycle and differentiate [76]. Chick retinal progenitor cells express both TRα, and at certain times, Dio3, the TH-degrading enzyme. One possibility is that T3 levels are kept low so that cycling progenitor cells don't prematurely make postmitotic daughters. One interpretation of the rGH-PAL data is that there could be competition for TRE binding sites by non-T3 responsive factors within a cycling cell, to ensure there isn't promiscuous activation of T3-sensitive genes. This is interesting to consider since, in the chick, TRβ can be turned on in late G2 of the cell cycle right before the cell divides, to create a TRβ+ postmitotic daughter cell [41]. Limiting the ability of TRα to activate transcription in progenitor cells might be one mechanism by which TRβ is only turned on when a progenitor cell is about to make a daughter which will terminally differentiate. The timing of this step might be regulated by availability of T3. It is interesting to note however, that in other situations, TH can induce proliferation, including within the Xenopus retina during metamorphosis [77], and in prostate carcinoma cells [78]. Its role in the Xenopus retina highlights its role during metamorphosis, where it acts as a developmental timer [77], [79]. THs role as a developmental timer is further demonstrated in keratinocytes, where proliferation is promoted by Shh directly inducing Dio3-controlled TH degradation [34].

### T3 addition drives reporter activity in the developing photoreceptor layer

T3 addition drove reporter activity in the ONL for the aTRE, rME, and DR4 reporters (Fig. 5F, 6F, 7F, 8F). In the case of the 1xDR4, ventral activation occurred primarily in the ONL, whereas in the dorsal retina both ONL and progenitor cells were labeled. Similar cell morphologies were labeled in the absence of exogenous T3. TRβ is also expressed in the ONL and in the mouse has been shown to be required for proper regulation of expression of blue and green opsin in developing photoreceptors [14]. As predicted, TRβ knock-down reduced both aTRE and rME activity (Fig. 5H, 6H). TRα mRNA is expressed at low levels throughout the radial dimension of the retina, most likely in both cycling progenitor cells and in postmitotic cells. Interestingly, TRα knock-down also substantially reduced aTRE and rME expression (Fig. 5G, 6G). One model is that reduction of TRα in the cycling progenitor reduces the ability of the progenitor to produce TRβ in late G2 and/or to produce a PR cell. TRα reduction would thus lead to less TRβ available for aTRE and rME activation in the ONL. Another model could have TRα directly driving TRβ expression in the newly postmitotic cells. Ubiquitous TRα RNA and ONL-specific TRβ RNA localization, together with localized TRE reporter activity in the TRβ+ ONL domain, predict that TRα may be upstream of TRβ in the chick retina. Consistent with this hypothesis, knock-down of TRα led to reduced 1xDR4 reporter activity in the central and ventral retina where T3 addition drove 1xDR4 activation in the ONL (Fig. 7G' compared to Fig. 7D'). TRβ knock-down didn't result in as much of a loss of activation in high T3 relative to TRα knock-down (Fig. 7H' compared to 7D'). However, the 2xDR4 element, which displayed robust ONL-localized T3-dependent activity and minimal activity in the absence of T3, displayed reduced central activity with both TRα and TRβ knock-down (Fig. 8G', 8H' compared to D'). Interestingly, the DR4 elements and TR-shRNA's reveal several unique domains of activity with a central domain dependent on TR+T3 for activation, a ventral domain that in the absence of T3 is repressed by the TRs, and dorsal domain that is TR-independent.

### TREs reveal D/V patterns in the absence of exogenous T3

**Figure 13:**
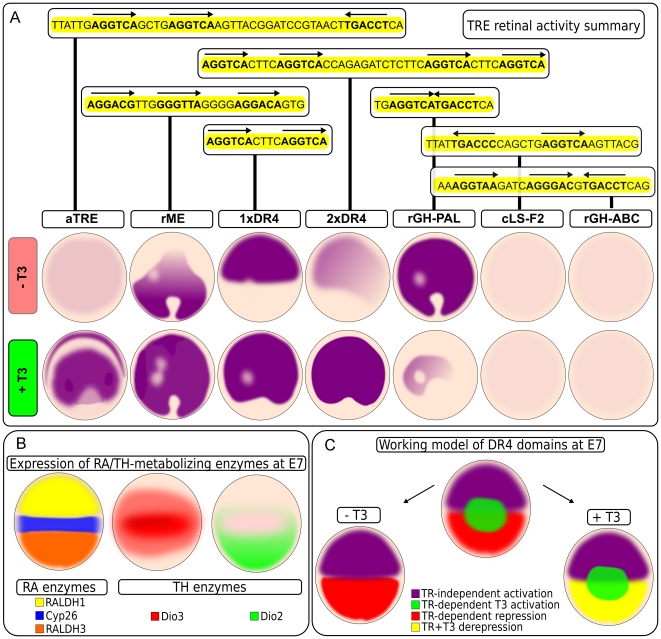
Summary of whole-mount TRE reporter patterns, schematic of RA/TH-metabolizing enzymes, and DR4 working model. A. Summary of TRE reporter patterns ±T3. B. Schematic of expression of RA and TH metabolizing enzymes at E7 time point. Expression of RA metabolizing enzymes RALDH1 (labeled yellow) and RALDH3 (RA-activating)(labeled orange) and Cyp26 (RA-degrading)(labeled blue) enzymes. Expression of Dio3 (TH-degrading)(labeled red) enzyme. Expression of Dio2 (TH-activating)(labeled green) enzyme. C. DR4 working model. The DR4 element is activated in three discrete domains dorsally, centrally, and ventrally through TR-dependent and TR-independent mechanisms. In the absence of T3, the model predicts a TR-independent activation dorsally and TR-dependent repression ventrally. In the presence of T3, the model predicts a ventral TR+T3 de-repression, central TR+T3 activation, and dorsal TR-independent activation.

Some of the most interesting results revealed by TRE activation were the striking patterns that were observed in low T3 conditions (summarized in Fig. 13A), most notably the ventral rME activity (Fig. 6A) and the dorsal 1xDR4 activity (Fig. 7A). The whole mount rME pattern is similar to that of Dio2 (TH-activating enzyme) and RALDH3 (RA-activating enyzme) RNA expression at E7 (Fig. 13B). These patterns are also reminiscent of much later rhodopsin RNA expression, which is expressed in rod photoreceptors [71]. The rME activity is localized primarily to the developing ONL, as are both Dio2 and PR TRβ RNA [41]. As both TRα and TRβ shRNA reduce rME activity (Fig. 6G, 6H), it is not yet clear if ONL cells express TRα at low levels or if TRα knock-down is reducing the TRβ level, which is the receptor presumably driving rME activity in the ONL.

The whole mount 1xDR4 pattern of dorsal activation (Fig. 7A) closely resembles that of RALDH1 (RA-activating enzyme) RNA expression (Fig. 13B). However, unlike the rME, dorsal 1xDR4 activity localized to both the ONL as well as the progenitor zone. Furthermore, T3 addition increased 1xDR4 activity in both the ONL and GCL. Dimerizing the DR+4 element substantially reduced the ONL and ONBL cells in the absence of T3 and resulted in heavy labeling of the ONL in high T3, in a similar pattern to that of rME +T3. Restriction of the dimerized DR4 to only the ONL supports the idea that TRβ may be able to more efficiently bind the 2xDR4 at the expense of TRα and suggests that the 2xDR4 may be more sensitive to TRβ activation. However, the dorsalized activity of the 1xDR4 in the absence of T3, the difference in activity and cell types labeled between 1xDR4 and 2xDR4, as well as the double-shRNA DR4 activity, are all difficult to explain without invoking multiple and complex models. Nonetheless, if one examines the ventral, central, and dorsal domains of DR4 activity independently, it becomes possible to build a model consistent with these data. These models include the activity of non-TRs on some of these reporters, as well as competition for some TREs between non-TRs and TRs, and potentially homeostatic regulation of the TH pathway. Such models will require additional experiments for validation.

### TREs reveal 3 domains in the retina

Analysis of TRE activity during retinal development offers a glimpse of the complexity that multiple transcription factors, acting even on a relatively simple element(s), can play during development. There is expression of the synthetic RA enzymes, and presumably signaling, in the dorsal and ventral retina [80], [81], [28]. Additionally, there is expression of a RA degrading enzyme (Cyp26) in the central retina that is presumably devoid of RA signaling (Fig. 13B) [80], [28]. Dio3 (TH-degrading enzyme) expression overlaps with this Cyp26 domain at one particular time point (Fig. 13B). Similarly, Dio2 (TH-activating enyzme) expression begins within this Cyp26 domain, but is excluded from the presumptive central rod-free zone and primarily expands only ventrally (Fig. 13B). Expression of these signaling components is developmentally dynamic and at any point in development the combinatorial intersection of these components define unique domains. Dorsally, this domain would be defined as RALD1+, Dio3+, Dio2- (high RA activity, low TH activity). Centrally, this domain would be defined as Cyp26+, Dio3+, Dio2- (low RA activity, low TH activity). The ventral domain would be defined as RALDH3+ with a wave of Dio2 and Dio3 passing through (high RA activity, pulse/chase of TH activity).

The D/V patterning of the 1xDR4 reporter in low T3 suggests that it may be reading out both TH and RA signaling pathways. The whole mount activation patterns of both the 1xDR4 and rME in low T3 correspond with domains of high RA component expression (and presumably RA signaling). Additionally, the dorsal 1xDR4 activation domain (in the absence of added T3) should have overall low TH activity, whereas the rME activation domain should have higher TH activity, as predicted by the ventral expression pattern of Dio2 [41]. T3 addition to the high RA/low TH dorsal domain resulted in 1xDR4 activation in both progenitor and ONL cells, whereas T3 addition to the high RA/TH pulse domain in the ventral retina resulted in stronger activation only in ONL cells. As Dio3 and Dio2 are expressed by different cell types, and Dio2 expression is predominately ventral and in the ONL, these observations suggest that T3 addition may overwhelm Dio3 activity in progenitor cells dorsally such that there is activation in progenitor cells as well as more activity in the ventral postmitotic ONL cells. One model might be that, in a low T3 environment, RA signaling activates the 1xDR4 dorsally while the TRs + low T3 repress 1xDR4 ventrally (Fig. 13C). Loss of both TRs in low T3 results in ventral de-repression and allows for mild ventral activation through the RA pathway. High T3 results in activation in both the ventral and central retina by the TRs, with TRα playing a more prominent role in T3-dependent central activation and TRβ being more important for mediating ventral repression in the absence or low levels of T3 (Fig. 13C).

The central low RA/low TH domain seems to have a unique property in that it is resistant to reporter activation. Several reporters, under different T3 conditions and with varying TR-shRNA perturbations, revealed a specific nasal/central AP-negative patch. Strikingly, the rGH-PAL, which was repressed by TR+T3, and the rME and DR4 TREs, which were activated by TR+T3, all displayed this nasal/central AP-negative patch independent of their T3 response state. The patch became more prominent with DR4 elements upon double TR-shRNA perturbation in the absence of T3. Interestingly, this patch corresponds in location to the developing rod-free zone [71]. This may indicate that, in addition to low RA and low TH signaling, there is expression of a repressor that prohibits NR activation in the restricted domain that will ultimately develop into the rod-free zone. The importance of this is presently unclear and remains to be elucidated.

### Deiodination, TH transport, and signaling implications

It was satisfying to observe that activation of several reporters was TH-sensitive in a highly localized manner in the developing ONL. Localized ONL expression is consistent with previous work in mouse and fish highlighting the importance of local TH levels, developmental timing of TH exposure, and especially TRβ expression in cone opsin regulation and photoreceptor maturation [14], [42]–[44], [46], [48], [49]. In the brain, Dio2 is expressed predominately by glial cells whereas neurons express Dio3 and TRs, and are presumably the target cell for locally generated T3 [51]–[57]. An alternate model is that neurons use unliganded TRs and Dio3 to keep TH sensitive genes off, and that glial Dio2-generated T3 functions cell autonomously. Current understanding of cellular transport of TH, as well as autonomous versus non-autonomous TH signaling in the CNS, is incomplete at best. In contrast to the brain, during development of the chick retina a subset of postmitotic neurons, not glia, express Dio2. It is currently unclear if the T3 generated from these Dio2+ neurons is acting in a cell autonomous fashion or, as modeled for the brain, in a cell non-autonomous manner on either neighboring mitotic Dio3+ cells or newly postmitotic TRβ+/Dio2- cells. Interestingly, the monocarboxylate TH transporter-8 (MCT8) is expressed through the radial dimension of the chick retina in a ubiquitous pattern similar to that of TRα (data not shown). A major strength of the TRE reporter approach is that it is now possible to read out TR-dependent T3-sensitive reporter activity in a cell specific manner during development. TRE reporters combined with selective shRNA knock-down of the deiodinase components will provide a powerful tool for unraveling the complexities of *in vivo* TH homeostasis, autonomous versus non-autonomous deiodinase signaling, and early TR-dependent developmental events. The unexpected TH-independent activation and TR-dependent repression of reporters in retinal progenitor cells highlights the complexity of transcription regulation during development.

Overall, this study illustrates that previously-validated TREs can drive unique patterns of activity in both a TR-dependent and TR-independent fashion in the retina and illuminated the previously under-appreciated dynamic, complex, and competing nature of nuclear receptor signaling in a developing tissue. The data presented here support the possibility that nuclear receptor competition for particular sites provides a potential intersection among various nuclear receptors and their ligands, including TH and RA. Regulation of ligand synthesis, destruction, and transport, as well as NR presence or absence, along with competition among cofactors, provide for multiple levels of regulation of target genes.

## Materials and Methods

### Plasmids

CAG-GFP and CAG-AP plasmids were from Matsuda and Cepko [68]. The pβactin-RFP-shRNAC chicken U6 plasmid was a gift from Wilson, S (University of Sheffield, UK) [70]. The mTRα and rTRβ over-expression plasmids were a generous gift from Larsen, PR (Harvard Institute of Medicine, USA) [82], [83]. The TRE reporters were constructed using the GFP-IRES-PLAP plasmid backbone from Kim et al. [84] which was further modified with a polyA terminator [85], MCS, and a minimal 15 base TATATAATGGAAGCT promoter driving eGFP and an IRES-PLAP (Stagia3). Stagia3 stands for Stop TAta eGfp Ires Ap version 3. The primary Stagia3 sequence is available in Supporting Text File S1. TREs were constructed by annealing complimentary oligos together such that correct annealing generated the appropriate TRE flanked by a 5' XhoI and 3' EcoRI sites. The XhoI/EcoRI TRE fragment was then cloned into the MCS of the Stagia3. The CAG-EGFP-IRES-PLAP plasmid was constructed by cloning the broadly expressed CAG promoter upstream of the TATA sequence in the Stagia3 vector [69].

### Chicken shRNA constructs

Three target shRNA sequences were generated for each gene using the Genescript siRNA Target Finder tool (www.genscript.com/tools.html). Chicken shRNA cassettes were built as previously described [70] with each shRNA sequence inserted using NheI/MluI sites. shRNA's were validated in 293T cell culture transfections with sensor constructs consisting of a CAGs promoter driving target sequences followed by an IRES-eGFP [68] (data not shown). GFP fluorescence was monitored at 48 hours to assess shRNA knock-down efficiency. The TRα (NM_205313) target shRNA sequences are as follows:

shRNA #1 GCAAGTCGCTGTCTGCCTTCAA (1190-1211)

shRNA #2 GGCTACCACTACCGCTGCATCA (466-487)

shRNA #3 AGTGCCAGCTGTGCCGCTTCAA (599-620).

The TRβ (NM_205447) target shRNA sequences are as follows:

shRNA #1 GAGTGAGACTTTAACGCTAAAT (923-944)

shRNA #2 GCAGTTGTATCACTGGATGAAA (1497-1518)

shRNA #3 CAGTCCTTTGATTGAGCAAATA (1575-1596).

The GAPDH (NM_204305) target shRNA sequences are as follows:

shRNA #1 TGATAGAAACTGATCTGTTTGT (1228-1249)

shRNA #2 TCTGTTTGTGTACCACCTTACA (1241-1262)

shRNA #3 GAAACTGATCTGTTTGTGTACC (1233-1254).

### *In vitro* electroporation

The right retinae of E5 chicken embryos (Hamburger-Hamilton stage 25 to stage 26) were dissected in warm DMEM/F12 50/50 mix, removing the RPE but leaving the lens. Using the BTX Electro Square Porator ECM830 three retinae were simultaneously electroporated in a 150 µl chamber containing plasmid DNA (200 ng/µl of each construct final concentration) with 25 mV for five cycles of 50 mseconds pulse and 950 mseconds chase. Retinae were placed in the chamber lens-side facing the positive electrode and with the central retina facing the negative electrode so that the dorsal and ventral retina were electroporated equally. The lens was then removed and the retina cultured as an explant on a floating Nuclepore Track-Etch membrane (Whatman, 110606) in DMEM with 10% (v/v) charcoal-stripped fetal calf serum (FBS), 100 U/mL penicillin (Invitrogen), 100 mg/mL streptomycin (Invitrogen), and with ±100 nM T3 (Sigma) added.

### Cell culture and polyethylenimine transfections

HEK293 (human embryonic kidney cell line) and 293T were routinely grown in Dulbecco's Modified Eagle's medium (DMEM) supplemented with 10% (v/v) FBS, 100 U/mL penicillin (Invitrogen), and 100 mg/mL streptomycin (Invitrogen). Cells treated ±T3 were cultured in DMEM with 10% charcoal-stripped FBS, 100 U/mL penicillin (Invitrogen), 100 mg/mL streptomycin (Invitrogen), and varying concentrations of T3. Cells were transfected using 1 mg/ml polyethylenimine (PEI) [Polyscience, Inc. cat 23966] in a ratio of 4 µl PEI per 1 µg DNA [86]. 1 ug of total DNA (333 ng receptor/333 ng TRE/333 ng RFP) +100 µL serum-free DMEM were first mixed. 8 µL of PEI was then added and the mixture vortexed and then left at room temperature for 15 minutes. Old media was then removed from the plate and new media added. The PEI/DNA mixture was then added to the side of the plate, shaking the plate gently by hand to mix. Transfected cells were subsequently incubated for 24 h±T3 (Sigma, St. Louis, MO) in various concentrations. After 24 hours, cells were first analyzed for RFP and GFP fluorescence, and then developed for AP detection.

### Histochemical AP staining

Cells were washed in 1xPBS to remove residual media and then fixed for 5 minutes in 4% paraformaldehyde (PFA) in PBS. Cells were then washed three times in 1xPBS, pH 7.4, and floated in a 65°C water bath for 1 hour 15 minutes to heat-inactivate endogenous AP activity. Cells were then placed in nitroblue tetrazolium (NBT)(Sigma) and the AP was developed for 15 minutes using NBT and 5-bromo-4chloro-3-indolyl-phosphate (BCIP)(Sigma) for color detection. After 15 minutes, cells were washed in 1xPBS and imaged immediately. Whole mount explanted retinae were first washed in 1xPBS and then fixed in 4% PFA for 30 minutes at room temp. Retinae were then washed several times in 1X PBS prior to heat-inactivating for 1 hour 15 minutes at 65°C in 1X PBS. Retinae were then placed in NTM pH 9.5 (100 mM NaCL, 100 mM Tris pH 9.5, 50 mM MgCl_2_) and the AP was developed slowly for 15 hours at 4°C overnight using NBT and BCIP for color detection.

### Preparation of retinal sections

For experiments in which NBT/BCIP staining was not performed, retinaes were removed from culture, fixed in 4% PFA for 30 minutes at room temperature, and washed three times in 1xPBS. Retinae were cryoprotected in 30% sucrose overnight or until the tissue sank. The tissue was then equilibrated in a 50/50 mix of 30% sucrose and OCT compound (Tissue-Tek 4583) for 1 hour. The retinae were then carefully orientated, using the ventral fissure as a reference landmark, and embedded in OCT blocks for cryosectioning. Frozen sections were cut at 20 microns on a Leica CM3050 S cryostat.

### Immunohistochemistry

Retinal sections were stained with antibodies as previously described [87]. PLAP antibody was a monoclonal Anti-Alkaline Phosphatase, Human Placental antibody (Sigma A2951) (1∶500). Secondary antibody was Goat anti-mouse Cy3 (1∶500).

### Image analysis

Cell culture experiments were imaged with a Leica DMI3000B inverted microscope and a Leica DC200 camera. All wells on one plate transfected with one set of plasmids were imaged on the same day with exposures of 1/200” (bright field optics [BF]) and 1/19” (GFP/RFP fluorescent optics). Whole-mount retinae were imaged with a Leica MZFLIII dissecting scope and a Nikon DXM1200F camera with exposures of 1/200” (BF) and 1”(RFP). Bright-field and epi-fluorescence sections were imaged using a Nikon Eclipse E1000 microscope and a Nikon DXM1200F camera. For GFP-IRES-PLAP co-localization, a 5.7 micron z-series was collected on 20 micron frozen sections with a Leica SP2 inverted confocal microscope. Maximum intensity projections were generated with Imaris-bitplane software (Bitplane Inc, Saint Paul, MN, USA).

### Software

Open source software, with the exception of the MIPs generated via Imaris, was used for all data analysis, figure generation, manuscript preparation, and referencing. Operating system used was Debian-based Ubuntu 9.10 and 10.04 (http://www.ubuntu.com/) running linux kernel v2.6.31-20 and v2.6.32-24. Scalable vector graphics were generated using Inkscape (http://www.inkscape.org/) and figure layouts were created using the GNU Image Manipulation Program v2.6.7 [GIMP] (http://ww.gimp.org/). Manuscript was written using OpenOffice writer v3.1.1 (www.openoffice.org/). Reference library was managed using Mozilla Firefox v3.5.9 (http://ww.mozilla.com/en-US/) and the Firefox Zotero extension v2.0.2 (http://www.zotero.org/). The Zotero reference library interfaced with OpenOffice writer via a word processor plugin v3.0a7 (http://www.zotero.org/support/word_processor_plugin_installation).

## Supporting Information

Figure S1 rGH-PAl, rGH-ABC, pCAG-GFP, and pCAG-AP tested in 293T cell-line. Each series of panels is labeled in the same order: A - RFP, A' - GFP, B - PLAP + either TRα or TRβ for the labeled TRE indicated ±T3. For each series, A and A' are the same field of view imaged, whereas B is a different field of view imaged. A-H. rGH-PAL tested for T3 response via TRα or TRβ in cell culture. rGH-PAL + TRα ±T3 (A-B, E-F). rGH-PAL + TRβ±T3 (C-D, G-H). I-P. rGH-ABC reporter tested for T3 responsiveness in cell culture. rGH-ABC + TRα±T3 (I-J, M-N). rGH-PAL + TRβ±T3 (K-L, O-P). Q-T. pCAG-GFP and pCAG-AP positive controls for GFP and AP detection. pCAG-GFP (Q, S) or pCAG-AP (R, T), and pβactin-RFP in the presence of 100 nM T3 (Q-R) or in the absence of exogenously added T3 (S-T). RFP fluorescence from pβactin-RFP +T3 (Q) or -T3 (S). GFP fluorescence from pCAG-GFP reporter +T3 (Q') or -T3 (S'). AP activity from pCAG-AP reporter +T3 (R) or -T3 (T).
(1.65 MB TIF)

Figure S2 AP immunoreactivity and GFP fluorescence correlate despite PLAP's position after the IRES Sequence. Embryonic day 5 retinas were electroporated with CAG-EGFP-IRES-PLAP and cultured for 2 days. The upper layer of the cultured retina was electroporated and the lower portion was not electroporated. A. Confocal z-stack of sections probed immunohistochemically for AP (red), nuclei stained with DAPI (blue) and GFP visualized by endogenous fluorescence (green). B. GFP fluorescence alone of retina in A. C. AP signal alone of retina in A. D. Confocal z-stack of sections treated as A, but with AP primary antibody left out. E. GFP fluorescence alone of retina in D. F. No AP antibody control signal alone of retina in D.
(2.03 MB TIF)

Figure S3 Ex vivo electroporation of control vector in explanted embryonic day 5 chick retina. A-D. Retinae were electroporated with control vector + pβactin-RFP/GAPDH-shRNA and cultured in varying T3 concentrations. AP activity reads out reporter activity and RFP fluorescence serves both as a co-electroporation marker as well as a marker of cells expressing the GAPDH-shRNA. AP staining quenches RFP fluorescence so high levels of AP result in lower visible RFP fluorescence. All AP reactions were develooped for the same amount of time (see materials and methods). In order to keep retinae intact during the electoporation and culture process, a small amount of RPE/ires tissue was left around the lens (brown tissue) and should be disregarded when comparing conditions (see Figure 5A, 5B, 5E). Control vector + GAPDH-shRNA -T3 (A, A'), 100 pM T3 (B, B'), 1 nM T3 (C, C'), and 100 nM T3 (D, D'). E-F. Cryosectioning of retina. The ONL is labeled with a purple bar, the ONBL is labeled with a green bar, and the GCL is labeled with a blue bar. 20 um cryosection of control vector + GAPDH-shRNA -T3 (E, E') and 100 nM T3 (F, F'). G-H. TRα and TRβ shRNA in 100 nM T3. Control vector in 100 nM T3 + TRα-shRNA (G, G') or TRβ-shRNA (H, H').
(2.39 MB TIF)

Figure S4 Assay of the rGH-ABC reporter in the developing chick retina. A-D. rGH-ABC + pβactin-RFP/GAPDH-shRNA in varying T3 concentrations. rGH-ABC + GAPDH-shRNA -T3 (A, A'), 100 pM T3 (B, B'), 1 nM T3 (C, C'), and 100 nM T3 (D, D'). E-F. Cryosectioning of retina. 20 um cryosection of rGH-ABC + GAPDH-shRNA -T3 (E, E') and 100 nM T3 (F, F'). G-H. TRα and TRβ shRNA in 100 nM T3. rGH-ABC in 100 nM T3 + TRα-shRNA (G, G') or TRβ-shRNA (H, H').
(2.53 MB TIF)

Text S1 Primary Stagia3 sequence. PA terminator from pTA-Luc 1-207; MCS - Sal,Mfe1,Xho1,EcoR1 207-230; TATA 230-236; EGFP(Clontech) 314-1034; EMCV IRES 1064-1625; Human Placental AP 1662-3536; Rabbit globin polyA 3562-4092; Amp 6480-5621;
(0.01 MB RTF)
