# Supplementary figures and images for: Analysis of Thyroid Response Element Activity during Retinal Development
Source: PLoS One. 2010 Oct 29;5(10):e13739. doi: 10.1371/journal.pone.0013739 (PMC2966421; doi:10.1371/journal.pone.0013739)

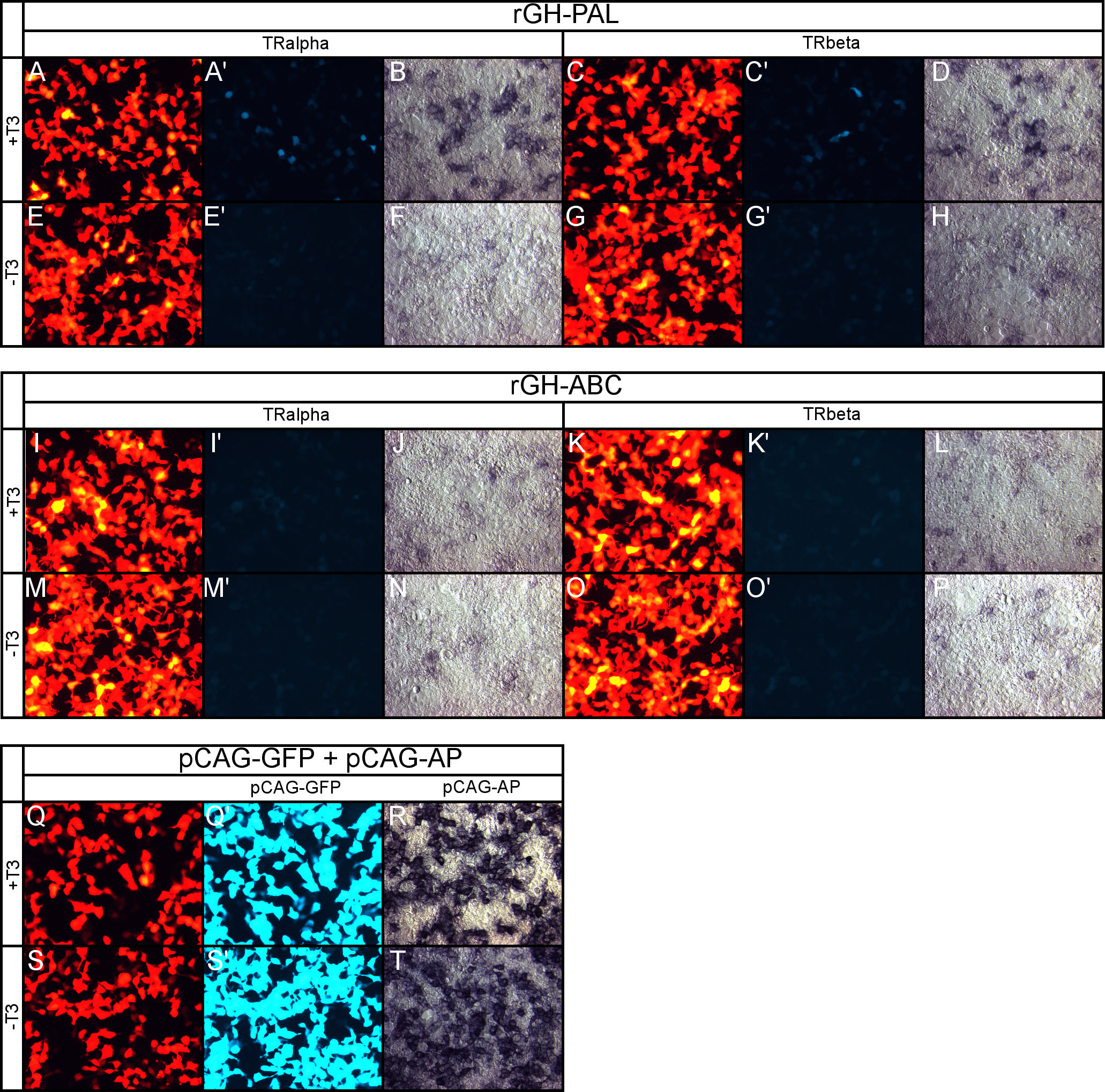

Supplement: Figure S1 — rGH-PAl, rGH-ABC, pCAG-GFP, and pCAG-AP tested in 293T cell-line. Each series of panels is labeled in the same order: A - RFP, A' - GFP, B - PLAP + either TRα or TRβ for the labeled TRE indicated ±T3. For each series, A and A' are the same field of view imaged, whereas B is a different field of view imaged. A-H. rGH-PAL tested for T3 response via TRα or TRβ in cell culture. rGH-PAL + TRα ±T3 (A-B, E-F). rGH-PAL + TRβ±T3 (C-D, G-H). I-P. rGH-ABC reporter tested for T3 responsiveness in cell culture. rGH-ABC + TRα±T3 (I-J, M-N). rGH-PAL + TRβ±T3 (K-L, O-P). Q-T. pCAG-GFP and pCAG-AP positive controls for GFP and AP detection. pCAG-GFP (Q, S) or pCAG-AP (R, T), and pβactin-RFP in the presence of 100 nM T3 (Q-R) or in the absence of exogenously added T3 (S-T). RFP fluorescence from pβactin-RFP +T3 (Q) or -T3 (S). GFP fluorescence from pCAG-GFP reporter +T3 (Q') or -T3 (S'). AP activity from pCAG-AP reporter +T3 (R) or -T3 (T). (1.65 MB TIF) [file pone.0013739.s001.tif]

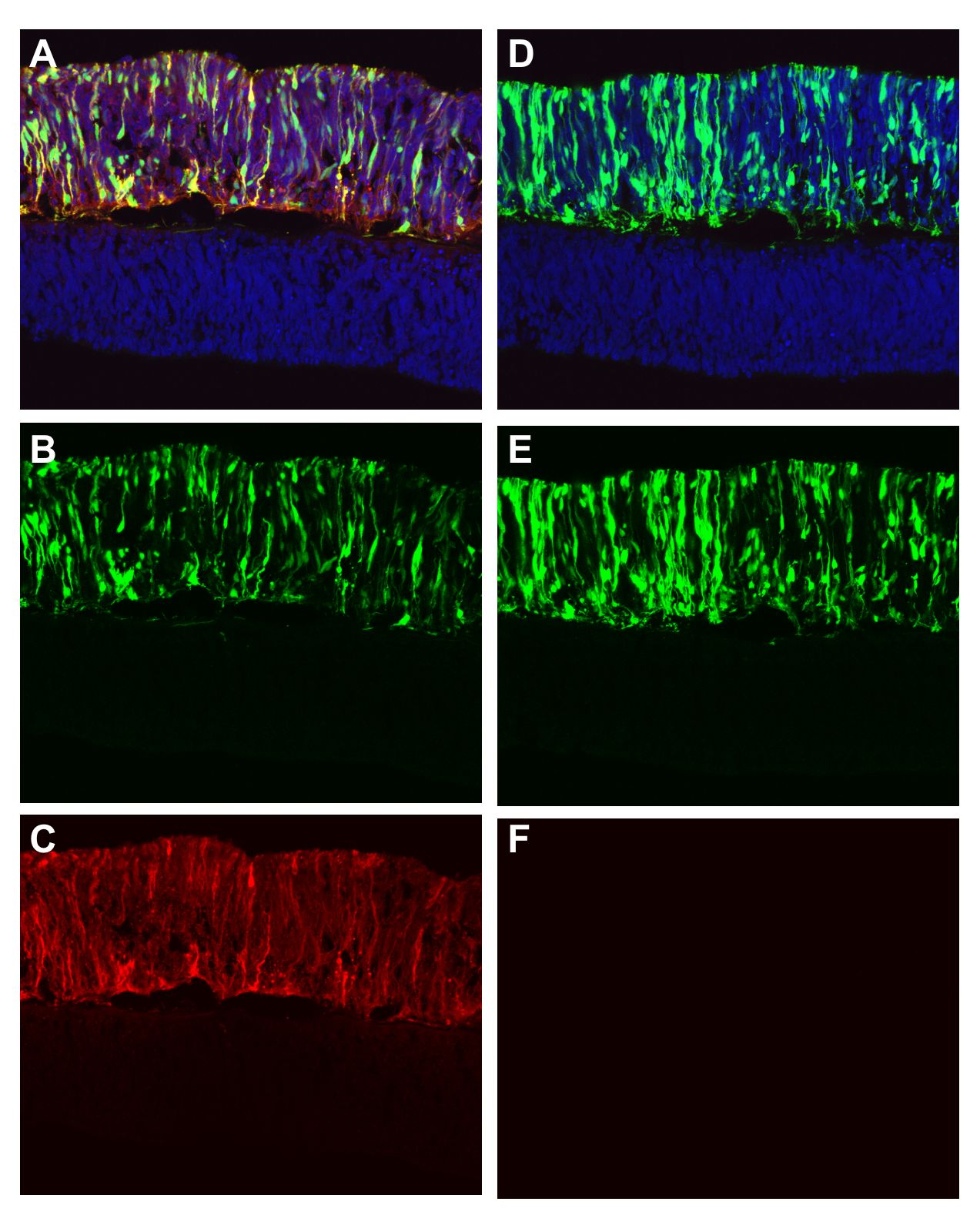

Supplement: Figure S2 — AP immunoreactivity and GFP fluorescence correlate despite PLAP's position after the IRES Sequence. Embryonic day 5 retinas were electroporated with CAG-EGFP-IRES-PLAP and cultured for 2 days. The upper layer of the cultured retina was electroporated and the lower portion was not electroporated. A. Confocal z-stack of sections probed immunohistochemically for AP (red), nuclei stained with DAPI (blue) and GFP visualized by endogenous fluorescence (green). B. GFP fluorescence alone of retina in A. C. AP signal alone of retina in A. D. Confocal z-stack of sections treated as A, but with AP primary antibody left out. E. GFP fluorescence alone of retina in D. F. No AP antibody control signal alone of retina in D. (2.03 MB TIF) [file pone.0013739.s002.tif]

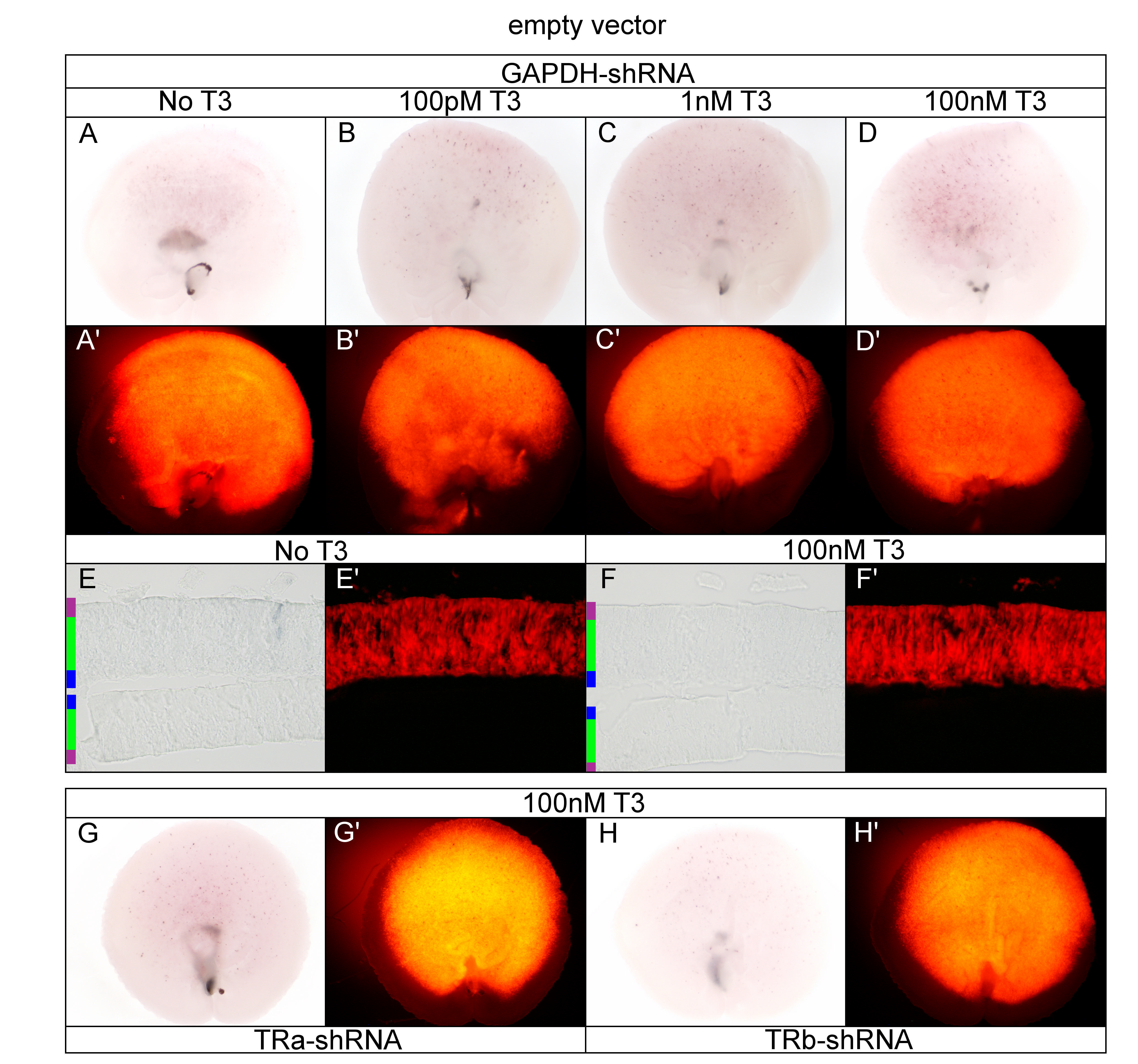

Supplement: Figure S3 — Ex vivo electroporation of control vector in explanted embryonic day 5 chick retina. A-D. Retinae were electroporated with control vector + pβactin-RFP/GAPDH-shRNA and cultured in varying T3 concentrations. AP activity reads out reporter activity and RFP fluorescence serves both as a co-electroporation marker as well as a marker of cells expressing the GAPDH-shRNA. AP staining quenches RFP fluorescence so high levels of AP result in lower visible RFP fluorescence. All AP reactions were develooped for the same amount of time (see materials and methods). In order to keep retinae intact during the electoporation and culture process, a small amount of RPE/ires tissue was left around the lens (brown tissue) and should be disregarded when comparing conditions (see Figure 5A, 5B, 5E). Control vector + GAPDH-shRNA -T3 (A, A'), 100 pM T3 (B, B'), 1 nM T3 (C, C'), and 100 nM T3 (D, D'). E-F. Cryosectioning of retina. The ONL is labeled with a purple bar, the ONBL is labeled with a green bar, and the GCL is labeled with a blue bar. 20 um cryosection of control vector + GAPDH-shRNA -T3 (E, E') and 100 nM T3 (F, F'). G-H. TRα and TRβ shRNA in 100 nM T3. Control vector in 100 nM T3 + TRα-shRNA (G, G') or TRβ-shRNA (H, H'). (2.39 MB TIF) [file pone.0013739.s003.tif]

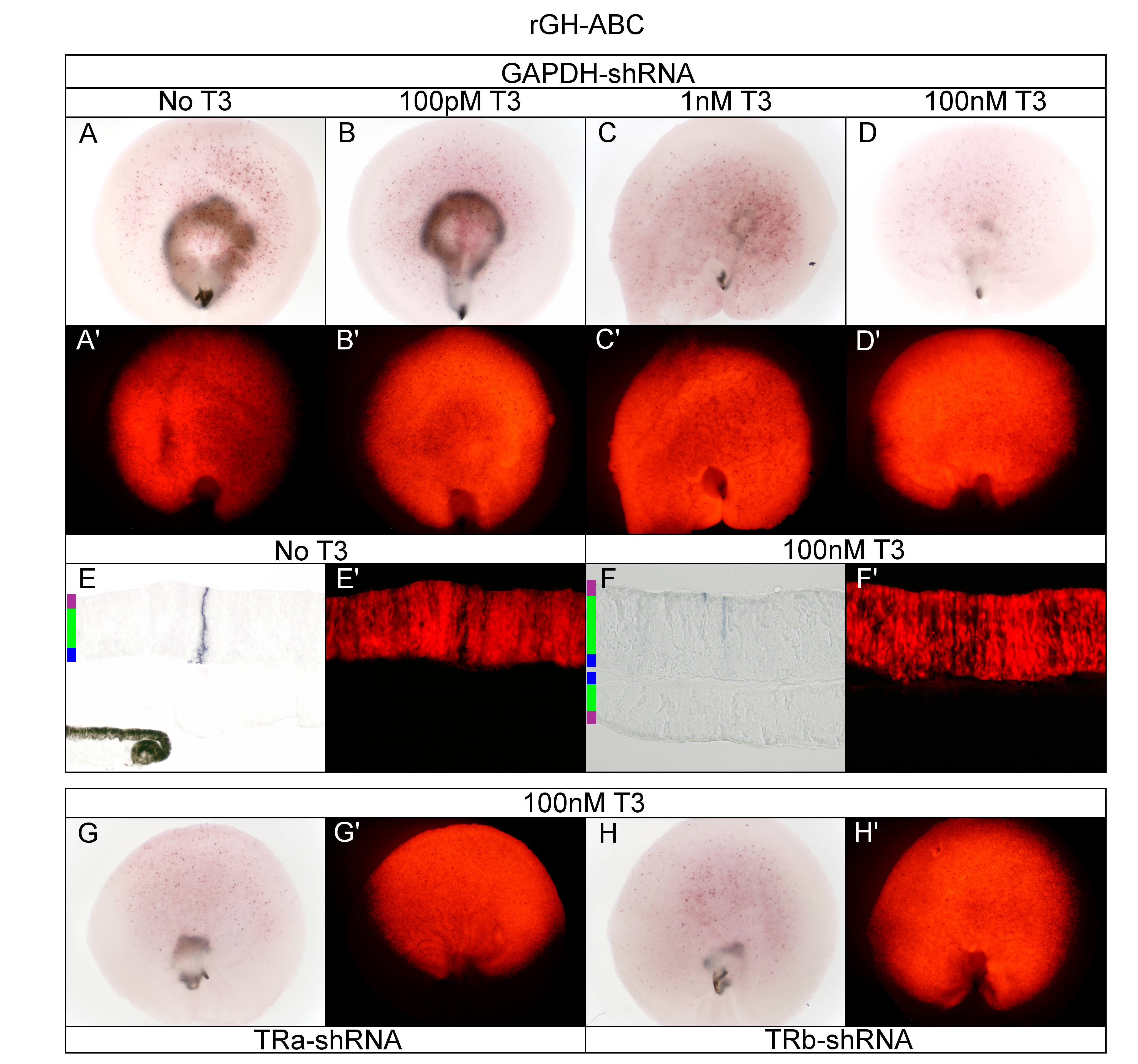

Supplement: Figure S4 — Assay of the rGH-ABC reporter in the developing chick retina. A-D. rGH-ABC + pβactin-RFP/GAPDH-shRNA in varying T3 concentrations. rGH-ABC + GAPDH-shRNA -T3 (A, A'), 100 pM T3 (B, B'), 1 nM T3 (C, C'), and 100 nM T3 (D, D'). E-F. Cryosectioning of retina. 20 um cryosection of rGH-ABC + GAPDH-shRNA -T3 (E, E') and 100 nM T3 (F, F'). G-H. TRα and TRβ shRNA in 100 nM T3. rGH-ABC in 100 nM T3 + TRα-shRNA (G, G') or TRβ-shRNA (H, H'). (2.53 MB TIF) [file pone.0013739.s004.tif]
