# Supplementary material for: Analysis of Thyroid Response Element Activity during Retinal Development
Source: PLoS One. 2010 Oct 29;5(10):e13739. doi: 10.1371/journal.pone.0013739 (PMC2966421; doi:10.1371/journal.pone.0013739)
Supplement: Text S1 — Primary Stagia3 sequence. PA terminator from pTA-Luc 1-207; MCS - Sal,Mfe1,Xho1,EcoR1 207-230; TATA 230-236; EGFP(Clontech) 314-1034; EMCV IRES 1064-1625; Human Placental AP 1662-3536; Rabbit globin polyA 3562-4092; Amp 6480-5621; (0.01 MB RTF) [file pone.0013739.s005.rtf]

Stagia3 sequence

PA terminator from pTA-Luc 1-207
MCS - Sal,Mfe1,Xho1,EcoR1 207-230
TATA 230-236
EGFP(Clontech) 314-1034
EMCV IRES 1064-1625
Human Placental AP 1662-3536
Rabbit globin polyA 3562-4092
Amp 6480-5621

GTCGAGAAGTAATATTAAGGTACGGGAGGTACTTGGAGCGGCCGCAATAAAATATCTTTATTTTCATTACATCTGTGTGT
TGGTTTTTTGTGTGAATCGATAGTACTAACATACGCTCTCCATCAAAACAAAACGAAACAAAACAAACTAGCAAAATAGG
CTGTCCCCAGTGCAAGTGCAGGTGCCAGAACATTTCTCTATCGATAGTCGACAATTGCTCGAGGAATTCTATATAATGGA
AGCTTGACCGGAGCCGTTAGTTGCCCGTCGGACGCGATTTGCGAGGCGCTTTATGCGCGCACCGGTCGCCACCATGGTGA
GCAAGGGCGAGGAGCTGTTCACCGGGGTGGTGCCCATCCTGGTCGAGCTGGACGGCGACGTAAACGGCCACAAGTTCAGC
GTGTCCGGCGAGGGCGAGGGCGATGCCACCTACGGCAAGCTGACCCTGAAGTTCATCTGCACCACCGGCAAGCTGCCCGT
GCCCTGGCCCACCCTCGTGACCACCCTGACCTACGGCGTGCAGTGCTTCAGCCGCTACCCCGACCACATGAAGCAGCACG
ACTTCTTCAAGTCCGCCATGCCCGAAGGCTACGTCCAGGAGCGCACCATCTTCTTCAAGGACGACGGCAACTACAAGACC
CGCGCCGAGGTGAAGTTCGAGGGCGACACCCTGGTGAACCGCATCGAGCTGAAGGGCATCGACTTCAAGGAGGACGGCAA
CATCCTGGGGCACAAGCTGGAGTACAACTACAACAGCCACAACGTCTATATCATGGCCGACAAGCAGAAGAACGGCATCA
AGGTGAACTTCAAGATCCGCCACAACATCGAGGACGGCAGCGTGCAGCTCGCCGACCACTACCAGCAGAACACCCCCATC
GGCGACGGCCCCGTGCTGCTGCCCGACAACCACTACCTGAGCACCCAGTCCGCCCTGAGCAAAGACCCCAACGAGAAGCG
CGATCACATGGTCCTGCTGGAGTTCGTGACCGCCGCCGGGATCACTCTCGGCATGGACGAGCTGTACAAGTAAAGCGGCC
AATTCTCCCTCCCCCCCCCCTAACGTTACTGGCCGAAGCCGCTTGGAATAAGGCCGGTGTGCGTTTGTCTATATGTTATT
TTCCACCATATTGCCGTCTTTTGGCAATGTGAGGGCCCGGAAACCTGGCCCTGTCTTCTTGACGAGCATTCCTAGGGGTC
TTTCCCCTCTCGCCAAAGGAATGCAAGGTCTGTTGAATGTCGTGAAGGAAGCAGTTCCTCTGGAAGCTTCTTGAAGACAA
ACAACGTCTGTAGCGACCCTTTGCAGGCAGCGGAACCCCCCACCTGGCGACAGGTGCCTCTGCGGCCAAAAGCCACGTGT
ATAAGATACACCTGCAAAGGCGGCACAACCCCAGTGCCACGTTGTGAGTTGGATAGTTGTGGAAAGAGTCAAATGGCTCT
CCTCAAGCGTATTCAACAAGGGGCTGAAGGATGCCCAGAAGGTACCCCATTGTATGGGATCTGATCTGGGGCCTCGGTGC
ACATGCTTTACGTGTGTTTAGTCGAGGTTAAAAAACGTCTAGGCCCCCCGAACCACGGGGACGTGGTTTTCCTTTGAAAA
ACACGATGATAATATGGCCAAATATGGGGTCGAGGCTGCTGCTGCTGCTGCTGCTGGGCCTGAGGCTACAGCTCTCCCTG
GGCATCATCCTAGTTGAGGAGGAGAACCCGGACTTCTGGAACCGCGAGGCAGCCGAGGCCCTGGGTGCCGCCAAGAAGCT
GCAGCCTGCACAGACAGCCGCCAAGAACCTCATCATCTTCCTGGGCGATGGGATGGGGGTGTCTACGGTGACAGCTGCCA
GGATCCTAAAAGGGCAGAAGAAGGACAAACTGGGGCCTGAGATACCCCTGGCCATGGACCGCTTCCCATATGTGGCTCTG
TCCAAGACATACAATGTAGACAAACATGTGCCAGACAGTGGAGCCACAGCCACGGCCTACCTGTGCGGGGTCAAGGGCAA
CTTCCAGACCATTGGCTTGAGTGCAGCCGCCCGCTTTAACCAGTGCAACACGACACGCGGCAACGAGGTCATCTCCGTGA
TGAATCGGGCCAAGAAAGCAGGGAAGTCAGTGGGAGTGGTAACCACCACACGAGTGCAGCACGCCTCGCCAGCCGGCACC
TACGCCCACACGGTGAACCGCAACTGGTACTCGGACGCCGACGTGCCTGCCTCGGCCCGCCAGGAGGGGTGCCAGGACAT
CGCTACGCAGCTCATCTCCAACATGGACATTGATGTGATCCTAGGTGGAGGCCGAAAGTACATGTTTCGCATGGGAACCC
CAGACCCTGAGTACCCAGATGACTACAGCCAAGGTGGGACCAGGCTGGACGGGAAGAATCTGGTGCAGGAATGGCTCGGC
GAACGCCAGGGTGCCCGGTACGTGTGGAACCGCACTGAGCTCATGCAGGCTTCCCTGGACCCGTCTGTGACCCATCTCAT
GGGTCTCTTTGAGCCTGGAGACATGAAATACGAGATCCACCGAGACTCCACACTGGACCCCTCCCTGATGGAGATGACAG
AGGCTGCCCTGCGCCTGCTGAGCAGACACCCCCGCGGCTTCTTCCTCTTCGTGGAGGGTGGTCGCATCGACCATGGTCAT
CATGAAAGCAGGGCTTACCGGGCACTGACTGAGACGATCATGTTCGACGACGCCATTGAGAGGGCGGGCCAGCTCACCAG
CGAGGAGGACACGCTGAGCCTCGTCACTGCCGACCACTCCCACGTCTTCTCCTTCGGAGGCTACCCCCTGCGAGGGAGCT
CCTTCATCGGGCTGGCCGCTGGCAAGGCCCGGGACAGGAAGGCCTACACGGTCCTCCTATACGGAAACGGTCCAGGCTAT
GTGCTCAAGGACGGCGCCCGGCCGGATGTTACCGAGAGCGAGAGCGGGAGCCCCGAGTATCGGCAGCAGTCAGCAGTGCC
CCTGGACGAAGAGACCCACGCAGGCGAGGACGTGGCGGTGTTCGCGCGCGGCCCGCAGGCGCACCTGGTTCACGGCGTGC
AGGAGCAGACCTTCATAGCGCACGTCATGGCCTTCGCCGCCTGCCTGGAGCCCTACACCGCCTGCGACCTGGCGCCCCCC
GCCGGCACCACCGACGCCGCGCACCCGGGGCGGTCCGTGGTCCCCGCGTTGCTTCCTCTGCTGGCCGGGACCCTGCTGCT
GCTGGAGACGGCCACTGCTCCCTGAGTGTCCCGTCCCTGGGGCTCCTGCTTCCCCATCCCGGAGTTCTCCTGCTCCCCAC
CTCCTGTCGTCCTGCCTGGCCTCCAGCCCGAGTCGTCATCCCCGGAGTCCCTATACAGAGGTCCTGCCATGGAACCTTCC
CCTCCCCGTGCGCTCTGGGGACTGAGCCCATGACACCAAACCTGCCCCTTGGCTGCTCTCGGACTCCCTACCCCAACCCC
AGGGACTGCAGGTTGTGCCCTGTGGCTGCCTGCACCCCAGGAAAGGAGGGGGCTCAGGCCATCCAGCCACCACCTACAGC
CCAGTGGGTACGGTCGAGGATATCGCGGCCGGCCGCACTCCTCAGGTGCAGGCTGCCTATCAGAAGGTGGTGGCTGGTGT
GGCCAATGCCCTGGCTCACAAATACCACTGAGATCTTTTTCCCTCTGCCAAAAATTATGGGGACATCATGAAGCCCCTTG
AGCATCTGACTTCTGGCTAATAAAGGAAATTTATTTTCATTGCAATAGTGTGTTGGAATTTTTTGTGTCTCTCACTCGGA
AGGACATATGGGAGGGCAAATCATTTAAAACATCAGAATGAGTATTTGGTTTAGAGTTTGGCAACATATGCCATATGCTG
GCTGCCATGAACAAAGGTGGCTATAAAGAGGTCATCAGTATATGAAACAGCCCCCTGCTGTCCATTCCTTATTCCATAGA
AAAGCCTTGACTTGAGGTTAGATTTTTTTTATATTTTGTTTTGTGTTATTTTTTTCTTTAACATCCCTAAAATTTTCCTT
ACATGTTTTACTAGCCAGATTTTTCCTCCTCTCCTGACTACTCCCAGTCATAGCTGTCCCTCTTCTCTTATGAAGATCCC
TCGACCTGCAGCCCAAGCTTGGCGTAATCATGGTCATAGCTGTTTCCTGTGTGAAATTGTTATCCGCTCACAATTCCACA
CAACATACGAGCCGGAAGCATAAAGTGTAAAGCCTGGGGTGCCTAATGAGTGAGCTAACTCACATTAATTGCGTTGCGCT
CACTGCCCGCTTTCCAGTCGGGAAACCTGTCGTGCCAGCGGATCCGCATCTCAATTAGTCAGCAACCATAGTCCCGCCCC
TAACTCCGCCCATCCCGCCCCTAACTCCGCCCAGTTCCGCCCATTCTCCGCCCCATGGCTGACTAATTTTTTTTATTTAT
GCAGAGGCCGAGGCCGCCTCGGCCTCTGAGCTATTCCAGAAGTAGTGAGGAGGCTTTTTTGGAGGCCTAGGCTTTTGCAA
AAAGCTAACTTGTTTATTGCAGCTTATAATGGTTACAAATAAAGCAATAGCATCACAAATTTCACAAATAAAGCATTTTT
TTCACTGCATTCTAGTTGTGGTTTGTCCAAACTCATCAATGTATCTTATCATGTCTGGATCCGCTGCATTAATGAATCGG
CCAACGCGCGGGGAGAGGCGGTTTGCGTATTGGGCGCTCTTCCGCTTCCTCGCTCACTGACTCGCTGCGCTCGGTCGTTC
GGCTGCGGCGAGCGGTATCAGCTCACTCAAAGGCGGTAATACGGTTATCCACAGAATCAGGGGATAACGCAGGAAAGAAC
ATGTGAGCAAAAGGCCAGCAAAAGGCCAGGAACCGTAAAAAGGCCGCGTTGCTGGCGTTTTTCCATAGGCTCCGCCCCCC
TGACGAGCATCACAAAAATCGACGCTCAAGTCAGAGGTGGCGAAACCCGACAGGACTATAAAGATACCAGGCGTTTCCCC
CTGGAAGCTCCCTCGTGCGCTCTCCTGTTCCGACCCTGCCGCTTACCGGATACCTGTCCGCCTTTCTCCCTTCGGGAAGC
GTGGCGCTTTCTCAATGCTCACGCTGTAGGTATCTCAGTTCGGTGTAGGTCGTTCGCTCCAAGCTGGGCTGTGTGCACGA
ACCCCCCGTTCAGCCCGACCGCTGCGCCTTATCCGGTAACTATCGTCTTGAGTCCAACCCGGTAAGACACGACTTATCGC
CACTGGCAGCAGCCACTGGTAACAGGATTAGCAGAGCGAGGTATGTAGGCGGTGCTACAGAGTTCTTGAAGTGGTGGCCT
AACTACGGCTACACTAGAAGGACAGTATTTGGTATCTGCGCTCTGCTGAAGCCAGTTACCTTCGGAAAAAGAGTTGGTAG
CTCTTGATCCGGCAAACAAACCACCGCTGGTAGCGGTGGTTTTTTTGTTTGCAAGCAGCAGATTACGCGCAGAAAAAAAG
GATCTCAAGAAGATCCTTTGATCTTTTCTACGGGGTCTGACGCTCAGTGGAACGAAAACTCACGTTAAGGGATTTTGGTC
ATGAGATTATCAAAAAGGATCTTCACCTAGATCCTTTTAAATTAAAAATGAAGTTTTAAATCAATCTAAAGTATATATGA
GTAAACTTGGTCTGACAGTTACCAATGCTTAATCAGTGAGGCACCTATCTCAGCGATCTGTCTATTTCGTTCATCCATAG
TTGCCTGACTCCCCGTCGTGTAGATAACTACGATACGGGAGGGCTTACCATCTGGCCCCAGTGCTGCAATGATACCGCGA
GACCCACGCTCACCGGCTCCAGATTTATCAGCAATAAACCAGCCAGCCGGAAGGGCCGAGCGCAGAAGTGGTCCTGCAAC
TTTATCCGCCTCCATCCAGTCTATTAATTGTTGCCGGGAAGCTAGAGTAAGTAGTTCGCCAGTTAATAGTTTGCGCAACG
TTGTTGCCATTGCTACAGGCATCGTGGTGTCACGCTCGTCGTTTGGTATGGCTTCATTCAGCTCCGGTTCCCAACGATCA
AGGCGAGTTACATGATCCCCCATGTTGTGCAAAAAAGCGGTTAGCTCCTTCGGTCCTCCGATCGTTGTCAGAAGTAAGTT
GGCCGCAGTGTTATCACTCATGGTTATGGCAGCACTGCATAATTCTCTTACTGTCATGCCATCCGTAAGATGCTTTTCTG
TGACTGGTGAGTACTCAACCAAGTCATTCTGAGAATAGTGTATGCGGCGACCGAGTTGCTCTTGCCCGGCGTCAATACGG
GATAATACCGCGCCACATAGCAGAACTTTAAAAGTGCTCATCATTGGAAAACGTTCTTCGGGGCGAAAACTCTCAAGGAT
CTTACCGCTGTTGAGATCCAGTTCGATGTAACCCACTCGTGCACCCAACTGATCTTCAGCATCTTTTACTTTCACCAGCG
TTTCTGGGTGAGCAAAAACAGGAAGGCAAAATGCCGCAAAAAAGGGAATAAGGGCGACACGGAAATGTTGAATACTCATA
CTCTTCCTTTTTCAATATTATTGAAGCATTTATCAGGGTTATTGTCTCATGAGCGGATACATATTTGAATGTATTTAGAA
AAATAAACAAATAGGGGTTCCGCGCACATTTCCCCGAAAAGTGCCACCTG
